# 2D MXene‐Integrated 3D‐Printing Scaffolds for Augmented Osteosarcoma Phototherapy and Accelerated Tissue Reconstruction

**DOI:** 10.1002/advs.201901511

**Published:** 2019-11-26

**Authors:** Shanshan Pan, Junhui Yin, Luodan Yu, Changqing Zhang, Yufang Zhu, Youshui Gao, Yu Chen

**Affiliations:** ^1^ State Laboratory of High Performance Ceramics and Superfine Microstructure Shanghai Institute of Ceramics Chinese Academy of Sciences Shanghai 200050 P. R. China; ^2^ School of Materials Science and Engineering University of Shanghai for Science and Technology Shanghai 200093 P. R. China; ^3^ Department of Orthopedic Surgery Shanghai Jiao Tong University Affiliated Sixth People's Hospital Shanghai 200233 P. R. China

**Keywords:** bone tumors, MXenes, photothermal therapy, scaffolds, tissue engineering

## Abstract

The residual of malignant tumor cells and lack of bone‐tissue integration are the two critical concerns of bone‐tumor recurrence and surgical failure. In this work, the rational integration of 2D Ti_3_C_2_ MXene is reported with 3D‐printing bioactive glass (BG) scaffolds for achieving concurrent bone‐tumor killing by photonic hyperthermia and bone‐tissue regeneration by bioactive scaffolds. The designed composite scaffolds take the unique feature of high photothermal conversion of integrated 2D Ti_3_C_2_ MXene for inducing bone‐tumor ablation by near infrared‐triggered photothermal hyperthermia, which has achieved the complete tumor eradication on in vivo bone‐tumor xenografts. Importantly, the rational integration of 2D Ti_3_C_2_ MXene is demonstrated to efficiently accelerate the in vivo growth of newborn bone tissue of the composite BG scaffolds. The dual functionality of bone‐tumor killing and bone‐tissue regeneration makes these Ti_3_C_2_ MXene‐integrated composite scaffolds highly promising for the treatment of bone tumors, which also substantially broadens the biomedical applications of 2D MXenes in tissue engineering, especially on the treatment of bone tumors.

## Graphical abstract

The rational design of 2D MXenes in tissue‐engineering biomedical field is reported, taking their unique characteristics of highly efficient near‐infrared‐triggered photothermal‐conversion performance and enhanced bone‐tissue regeneration capability, especially on combating bone tumors.

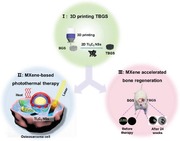

## 1 Introduction

Bone cancer is a general term for malignant bone tumors such as osteosarcoma, chondrosarcoma, and fibrosarcoma.^1^ It is usually divided into autologous skeletal system cancer and bone‐metastases cancer (primary tumors in breast, lung, and kidney).^2^ At present, the treatment of bone cancer usually combines destructive surgery (amputation or comprehensive limb salvage surgery) with multidrug chemotherapy, which has significantly improved the survival rate of patients.^3^ Unfortunately, the invasiveness of cancer as well as anatomical complexity determines an unattainable radical resection followed by inevitable local recurrence. Moreover, massive bone defects caused by surgery have surpassed the self‐healing ability of bone tissue, bringing long‐term pain to patients and even causing the failure of surgery.^4^ Consequently, it is highly urgent and necessary to construct multifunctional tissue‐engineering biomaterials with simultaneous bone‐tumor killing and bone‐tissue remodeling capacity.

Recently, ultrathin MXene nanosheets, as a new class of early transition metal carbides/nitrides/carbonitrides, have significantly enriched the 2D material families,^5^ which are featured with unique structural characteristics including large specific surface area and adjustable physiochemical property such as excellent electroconductibility. In 2D MXene, “M” denotes transition metal atoms, “X” means carbon or nitrogen, and “ene” suffix originating from “graphene” represents the materials with ultrathin 2D structure.^6^ For 2D MXenes' peculiarities, they have been broadly explored in versatile applications such as energy storage,^7^ catalysis,^8^ electromagnetic shielding,^9^ water purification,^10^ etc. The fast development of theranostic nanomedicine has promoted the extensive biomedical applications of these 2D MXenes in biosensing,^11^ intracellular fluorescent imaging,^12^ antibacterial,^13^ and photothermal therapy (PTT).^14^ Their fascinating biomedical performances promote the further extensive exploring of the unique and specific applications in versatile biomedical fields such as tissue engineering, which has not been achieved so far. 2D Ti_3_C_2_ MXenes possess high biocompatibility and desirable photothermal‐conversion efficiency in near‐infrared (NIR) biowindow.^15^ Especially, by the interaction of water and oxygen, they would degrade to release Ti‐based species, which is expected to promote the growth of new bones.^16^ Therefore, it is highly feasible to utilize the photothermal‐conversion property of 2D Ti_3_C_2_ MXene nanosheets (NSs) for ablating bone‐tumor cells, and then employ their biodegradable performance and biodegradation products for accelerating the bone reconstruction.

In general, the regrowth and regeneration of large bone defects still require some biomaterials to bridge the tissue gap and afford structural support to sustain the physiological activities and cellular behaviors during the new bone formation, such as nutrient transport, cell adhesion, proliferation, migration, differentiation, and maturation.^17^ Bioactive glass (BG) is a typical biomaterial for bone‐tissue regeneration,^18^ which has been demonstrated to be featured with high biocompatibility, osteoconductivity, osteoinductivity, and degradability.^19^ Therefore, the BG scaffolds (designated as BGSs) with 3D interconnected macropores, precisely controlled appearance and internal structures, as fabricated by the intriguing 3D‐printing technique, are generally regarded as the desirable candidate bridge biomaterial for hard‐tissue regeneration.^20^

**Scheme 1:**
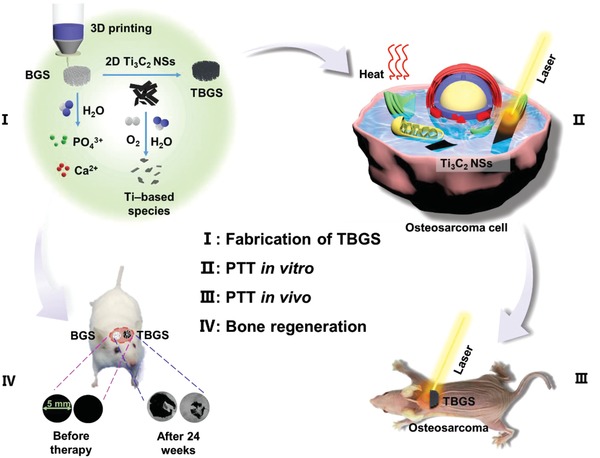
Schematic illustration of the fabrication of TBGS, ablation of bone cancer, and regeneration of bone tissue. I) Fabrication procedure of TBGS, including 3D printing of pure BGS, integration of Ti_3_C_2_ MXene, and degradation of Ti_3_C_2_ MXene on BGS. II, III) TBGS used for osteosarcoma cell elimination by photothermal ablation both in vitro (II) and in vivo (III). IV) Bone‐tissue reconstruction and the therapeutic results after the implantation of BGS and TBGS.

In this work, we report, for the first time, on the rational integration of 2D Ti_3_C_2_ MXenes with 3D‐printing BG scaffold (designated as Ti_3_C_2_‐BG scaffold or TBGS) for the construction of multifunctional biomaterial scaffold for bone‐cancer treatment with simultaneous bone‐tumor killing and bone‐tissue regeneration functionalities (**Scheme** **1**). On one hand, the integrated 2D Ti_3_C_2_ MXene NSs kill the bone cancer cells based on their specific photothermal‐conversion property. On the other hand, the implanted 3D BG component assists the differentiation of human bone marrow mesenchymal stem cells (hBMSCs) into osteoblasts by its bridging functionality.^21^ In the process of bone‐tumor treatment and bone‐tissue reconstruction, the titanium‐based species, as the biodegradation product from Ti_3_C_2_ MXene NSs, accelerate the formation of new bone.^16^, ^22^ During these activities, the BG component also gradually degrades to provide the necessary minerals and space for the newly formed bone tissue. Therefore, this rationally designed multifunctional 3D composite scaffold represents the novel therapeutic biomaterial for bone‐tumor therapy with concurrent cancer cell‐killing and tissue‐engineering performances.

## 2 Results and Discussion

### 2.1 Synthesis and Characterization of 2D Ti_3_C_2_ MXenes

**Figure 1:**
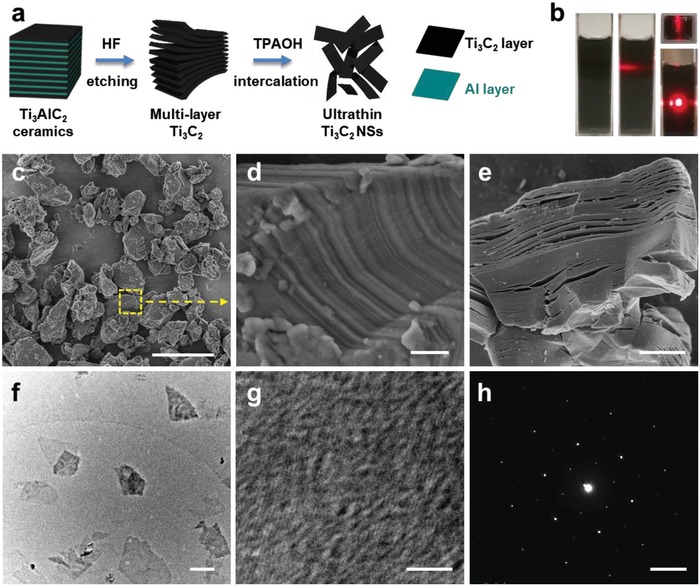
Synthesis and characterization of 2D Ti_3_C_2_ MXene NSs. a) Schematic illustration of the fabrication process of 2D Ti_3_C_2_ NSs, including HF etching and TPAOH intercalation of the original bulk Ti_3_AlC_2_ ceramic. b) Digital photographs of Ti_3_C_2_ NSs dispersed in aqueous solution. c) SEM image of bulk Ti_3_AlC_2_ ceramic (scale bar: 5 µm). d) Magnified SEM image of the selected area in (c) (scale bar: 200 nm). e) SEM image of multilayer Ti_3_C_2_ MXene after HF treatment (scale bar: 2 µm). f) TEM image of Ti_3_C_2_ NSs (scale bar: 200 nm). g) HRTEM image of Ti_3_C_2_ NSs (scale bar: 3 nm). h) SAED pattern of single‐layer Ti_3_C_2_ NSs (scale bar, 5 1/nm).

2D Ti_3_C_2_ MXenes, as a new photothermal nanoagent with excellent photothermal‐conversion property and high biocompatibility, were integrated with 3D‐printed BG scaffolds for killing bone cancer cells and regenerating massive bone defects. These 2D ultrathin Ti_3_C_2_ NSs were fabricated by hydrofluoric acid (HF) etching and subsequent tetrapropylammonium hydroxide (TPAOH) exfoliation of the original bulk MAX‐phase Ti_3_AlC_2_ ceramics (**Figure** **1**a).^23^, ^42^ The as‐prepared 2D Ti_3_C_2_ NSs could be well dispersed in aqueous solution as demonstrated by the obvious Tyndall effect (Figure 1b), enabling the further facile integration with 3D‐printing scaffolds.

The bulk Ti_3_AlC_2_ ceramic (MAX phase, Figure 1c,d) was initially etched by HF aqueous solution for 3 days to selectively remove the middle Al layer, which could fabricate multilayer Ti_3_C_2_ MXene with accordion‐like microstructure (Figure 1e).^9^ The elemental mapping of Ti_3_AlC_2_ ceramic reveal the co‐existence of Ti, Al, and C elements (Figure S1, Supporting Information), and the Al content of multilayer Ti_3_C_2_ was substantially decreased (Figure S2, Supporting Information) after HF etching. Subsequently, the etched Ti_3_C_2_ powder was intercalated with TPAOH solution for another 3 days to fabricate delaminated 2D ultrathin Ti_3_C_2_ NSs (Figure 1f). Both high‐resolution transmission electron microscopy (HRTEM, Figure 1g) image and the corresponding selected area electron diffraction pattern (SAED, Figure 1h) exhibit that the prepared 2D Ti_3_C_2_ NSs were featured with planar topology and hexagonal crystallize structure with well crystallinity, demonstrating the successful fabrication of 2D ultrathin 2D Ti_3_C_2_ MXene NSs.

### 2.2 Design, Fabrication, and Characterization of BG/Ti_3_C_2_‐BG Scaffolds

**Figure 2:**
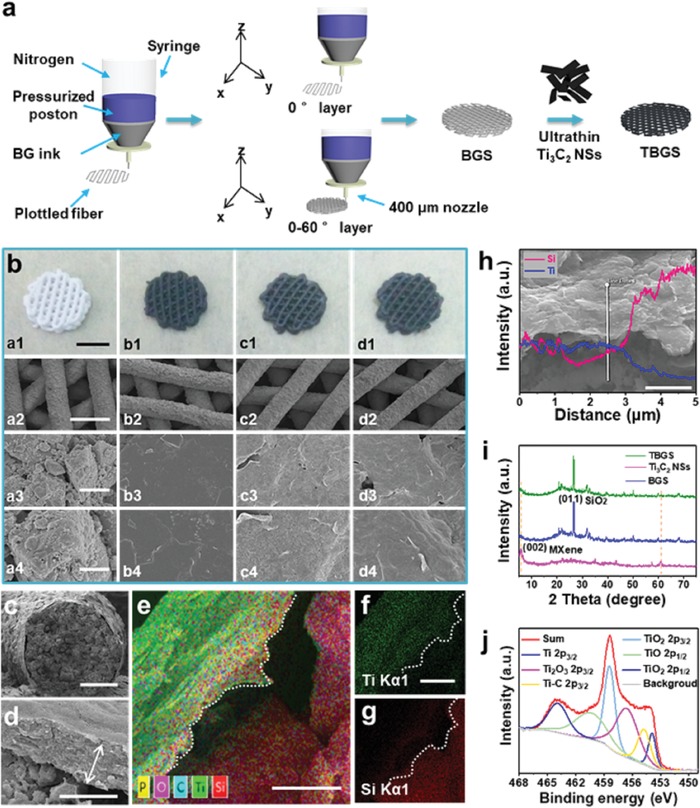
Fabrication and characterization of BGS/TBGS. a) Schematic illustration of the formation of pure BGS and TBGS, including 3D printing and further Ti_3_C_2_ MXene integration. b) Digital photographs and SEM images of pure BGS and TBGS: digital photographs of: a1) pure BGS, b1) 1.0 TBGS, c1) 1.5 TBGS, and d1) 2.0 TBGS; SEM images of: a2–a4) pure BGS, b2–b4) 1.0 TBGS, c2–c4) 1.5 TBGS, and d2–d4) 2.0 TBGS. From top to bottom, scale bars are 3 mm, 500 µm, 5 µm, and 1 µm, respectively. Each row of images shares the same scale bar. c,d) The fracture morphologies of 1.0 TBGS. Scale bar is 100 and 1 µm, respectively. e–g) Element mappings of 1.0 TBGS (all scale bars: 1 µm; e: merged image, f: Ti element, and g: Si element). h) SEM image and EDS (inset image) of 1.0 TBGS (scale bar: 3 µm). i) XRD patterns of Ti_3_C_2_ NSs, pure BGS, and TBGS. j) Ti 2p XPS spectra of TBGS.

**Figure** **2**a schematically depicts the formation of pure BGS by 3D‐printing technology.^24^ In this work, we applied a facile and efficient strategy, i.e., the direct solution‐soaking method, to prepare TBGS.^25^ To achieve a suitably modified amount for photothermal ablation, BGSs were integrated with Ti_3_C_2_ NSs at elevated initial concentrations (1.0, 1.5, and 2.0 mg mL^−1^). BGS integrated with 1.0 mg mL^−1^ Ti_3_C_2_ NSs was termed as 1.0 TBGS, and other TBGSs were renamed by this analogy. Digital photographs (Figure 2b‐a1–d1) reveal that the scaffolds fabricated by the 3D‐printing technique were featured with well‐designed microstructure, and the printed pure BGSs and TBGSs exhibited white color and black color, respectively. Scanning electron microscope (SEM) images (Figure 2b‐b2–d2) show that the Ti_3_C_2_ NSs modified the whole surface of BGSs and the corresponding structure was not compromised as compared to BGS (Figure 2b‐a2). Pure BGSs showed rough and loosened surfaces (Figure 2b‐a3,a4) while TBGSs had relatively smooth surfaces after the adsorption of nanosheets (Figure 2b‐b3–d3,b4–d4), which might be attributed to the planar structure of MXene covered onto the surface of BGSs.

Furthermore, the detailed fracture morphologies of 1.0 TBGS showed an obvious core–shell structure consisting of an ≈577 nm thick Ti_3_C_2_ shell and a BG core (Figure 2c,d). The element‐mapping analysis demonstrated the desirable element distribution, which exhibited that the Si, Ca, and P signals tended to increase from the surface to the interior, whereas the Ti and C signals tended to decrease (Figure 2e–g; Figure S3, Supporting Information). In addition, this tendency was also confirmed by the energy‐dispersive spectrometer (EDS) analysis (Figure 2h; Figure S4, Supporting Information), which provided the solid proof of coating a Ti_3_C_2_ MXene layer onto the surface microstructure of 3D BGS.

X‐ray diffraction (XRD) patterns of freeze‐dried Ti_3_C_2_ powder, BGS, and TBGS powders (Figure 2i) revealed the successful fabrication of Ti_3_C_2_ NSs and the effective integration of Ti_3_C_2_ NSs with BGSs. The distinct (002) peak in Ti_3_C_2_ powder could be ascribed to the 2D Ti_3_C_2_ MXene.^26^ The peak at 2θ ≈ 26.65° of BGS particles was indexed to the (011) planes of hexagonal SiO_2_ phase as the uppermost composition of BG, which was in accordance with the standard card of PDF#78‐1253. Both the (002) peak of Ti_3_C_2_ MXene and the (011) peak of BGS could be found in the diffraction peaks of TBGS, demonstrating the successful integration of Ti_3_C_2_ MXene and BGS. In addition, Raman spectra of Ti_3_AlC_2_, Ti_3_C_2_ NSs, BGS, and TBGSs powder are shown in Figure S5 in the Supporting Information. All of these consequences provided solid evidences that Ti_3_C_2_ NSs have been successfully adhered to BGSs from different aspects.

The surface status and chemical composition of BGSs and TBGSs were detected by X‐ray photoelectron spectroscopy (XPS). The characteristic peaks of BGSs were assigned to Si (Si 2p 103.3 eV), Ca (Ca 2p 347.5 eV), P (P 2p 133.5 eV), and O (O 1s 532.4 eV). The peak at the binding energy of 458.3 eV was assigned to Ti 2p of TBGSs, which indicates the existence of Ti_3_C_2_ NSs on the surface of modified BGSs (Figure S6, Supporting Information). Furthermore, the Ti 2p peak of TBGSs can be deconvoluted into six subpeaks (Figure 2j) at 453.9, 454.9, 456.9, 458.3, 460.2, and 464.2 eV corresponding to 2p_3/2_ (Ti), 2p_3/2_ (Ti–C), 2p_3/2_ (Ti_2_O_3_), 2p_3/2_ (TiO_2_), 2p_1/2_ (TiO), and 2p_1/2_ (TiO_2_), respectively. In addition to the inherent Ti—C bond of Ti_3_C_2_ NSs, XPS spectra demonstrated the presence of Ti and Ti*_x_*O*_y_*, revealing the Ti_3_C_2_ NSs were partially oxidized during the modification.

### 2.3 In Vitro Photothermal Performance, Cytotoxicity Assay, and Cell Ablation of Ti_3_C_2_‐BG Scaffolds

The essential characteristics of photothermal nanoagents for photonic tumor hyperthermia are the efficient optical absorption and high photothermal‐conversion efficiency in NIR biowindow.^27^ As illustrated in Figure S7a in the Supporting Information, the optical absorbance spectrum of Ti_3_C_2_ NSs in water presented a pronounced absorption in the range of 750–850 nm.^28^ Subsequently, we systematically assessed the effect of Ti_3_C_2_ concentrations, power density of laser irradiation, and the environment (dry and wet) on photothermal properties of the composite scaffolds after exposure to NIR laser irradiation.

**Figure 3:**
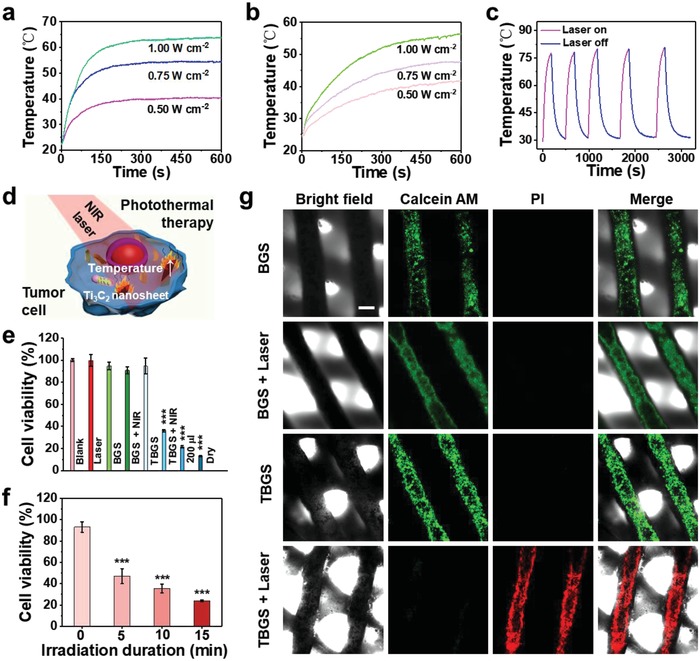
In vitro photothermal performance, cytotoxicity assay, and cell ablation of BGS/TBGSs. a) Photothermal‐heating curves of 1.0 TBGS under the irradiation with 808 nm laser at varied power densities (0.5, 0.75, and 1.0 W cm^−2^) in dry environment. b) Photothermal‐heating curves of 1.0 TBGS under the irradiation with 808 nm laser at varied power densities (0.5, 0.75, and 1.0 W cm^−2^) in wet environment. c) Heating curves of a TBGS for five laser on–off cycles (1.0 W cm^−2^) under irradiation with 808 nm laser. d) Schematic illustration for cancer‐cell ablation by Ti_3_C_2_ NSs possibly shedding from TBGS. e) Relative cell viability of bone‐tumor cells (Saos‐2) after the treatment with different conditions as described in the figure. The “200 µL” and “Dry” groups respectively mean TBGS +NIR group in 200 µL DMEM and dry environment (discarding all DMEM). The other groups were performed in 400 µL DMEM. The cell viabilities in “TBGS + NIR,” “200 µL,” and “Dry” groups were about 36%, 21%, and 13%, respectively. This result demonstrated that the increase of fluid could reduce the tumor‐killing effect of PTT. *n* = 4. f) Relative cell viability of Saos‐2 cells treated with 1.0 TBGSs for different irradiation durations. *n* = 4. g) CLSM images of live (green) and dead Saos‐2 cells (red) on BGSs or TBGSs with different treatments as indicated in the figure. Scale bar is 200 µm. *p < 0.05, **p < 0.01, ***p < 0.001.

Pure BGS and TBGSs at varied initial integrating concentrations (1.0, 1.5, and 2.0 mg mL^−1^) were exposed to an 808 nm laser irradiation for 10 min at a power density of 1.0 W cm^−2^ in the air (Figure S7b, Supporting Information). From 1.0 TBGS to 2.0 TBGS, the final equilibrium temperature increased from 55 to 65 °C within 10 min, while the temperature of pure BGS did not increase significantly. It indicated that the final equilibrium temperature and the heating rate of scaffolds were positively correlated with the integrated Ti_3_C_2_ amount. Furthermore, with the elevation of power density of irradiation laser from 0.5 to 1.0 W cm^−2^ (808 nm, 10 min), the equilibrium temperature of 1.0 TBGS increased from 40 to 65 °C in the air (**Figure** **3**a), and from 42 to 58 °C in phosphate buffer solution (PBS, Figure 3b), exhibiting that the photothermal effect of the composite TBGS was dependent on the power density of laser irradiation. The elevated temperature in PBS was slightly lower than the temperature in air because of the endothermic property of the liquid. Subsequently, the BGS and 1.0 TBGS were irradiated by 808 nm laser for 10 min at a power density of 1.0 W cm^−2^ (Figure S7c, Supporting Information). The temperature of TBGS increased by ≈20 °C in 10 min, but the pure BGS showed no obvious temperature variation.

To further investigate the photothermal stability of these MXene‐modified composite scaffolds, 1.0 TBGS was irradiated by 808 nm laser for ≈3 min (laser on) and then naturally cooled down to room temperature (laser off). After five “on–off” cycles of laser, the laser‐induced temperature increase showed no obvious deterioration (Figure 3c), indicating the high photothermal stability of these MXene‐integrated composite TBGSs for the potential continuous photothermal hyperthermia of bone tumors.

It has been demonstrated that the temperature around 45 °C could induce the tumor‐cell death.^29^ Based on the aforementioned photothermal evaluation results, 1.0 TBGS was chosen as the implanting composite scaffold for photothermal bone‐tumor hyperthermia. The in vitro cytotoxicity assay and cancer‐cell ablation of scaffolds were quantitatively evaluated by a standard Cell Counting Kit‐8 (CCK‐8) assay. Saos‐2 cells (osteosarcoma cells) were incubated with pure BGSs and TBGSs, which were further irradiated by 808 nm laser for triggering photothermal ablation (Figure 3d). As shown in Figure 3e, compared to the control group (blank), the percentages of viable cells in the BGS, BGS + laser, laser only, and TBGS groups ranged from about 90–100% with no significant difference, which manifested high biocompatibility of the fabricated TBGSs. Comparatively, less than 40% of Saos‐2 cells survived in the TBGS + laser group, revealing the capability of TBGS for efficiently killing cancer cells by photothermal ablation. Especially and importantly, with the prolonging of the laser‐irradiation duration (Figure 3f), times, and power density (Figure S7e,f, Supporting Information), there were distinctly much fewer living cells survived in the TBGS + laser group. For instance, the percentages of viable cells after laser irradiation for 15 min (1.0 W cm^−2^, one time), irradiation for three times (1.0 W cm^−2^, 10 min), and irradiation at 1.0 W cm^−2^ (10 min, one time) were about 25%, 25%, and 38%, respectively, demonstrating the high controllability of TBGS‐assisted photothermal ablation of cancer cells.

In addition, the Saos‐2 cell apoptosis after photothermal ablation was further intuitively confirmed by confocal laser scanning microscopy (CLSM) observations (Figure 3g). After laser irradiation (10 min, 1.0 W cm^−2^), the dead and live cells in scaffolds were specifically stained by propidium iodide (PI; red) and calcein‐AM (green), respectively. It is found that the experimental group (TBGS + laser) and control groups (BGS, BGS + laser, TBGS) exhibited a sharp contrast in fluorescence color where the experimental group showed the significant red fluorescence, indicating the effective cell apoptosis as induced by photothermal ablation. Furthermore, the number of apoptotic cells (Q1: dead cells + Q2: late apoptotic cells + Q4: early apoptotic cells) of flow cytometric analysis (Figure S8, Supporting Information) in group BGS, TBGS, BGS + NIR, and TBGS + NIR were determined to be 8%, 7%, 9.3%, and 43%, respectively. These results strongly demonstrated that TBGSs possessed powerful tumor‐cell ablation capacity under the irradiation of NIR laser in vitro. Especially, as could be seen from all bright‐field images (Figure 3g), each group of scaffolds still maintained a well‐ordered hierarchical 3D geometric structure after laser irradiation and further immersion scouring.

### 2.4 In Vivo Photothermal Tumor Ablation by MXene‐BG Scaffold under NIR Irradiation

Encouraged by in vitro excellent photothermal performance of TBGSs, a localized in vivo photothermal tumor ablation was scheduled. However, there are some critical challenges to establish an orthotopic osteosarcoma model for this multilevel research. First and foremost, leakage is a severe complication during intrafemoral/intratibial injection of tumor cells to establish the orthotopic model.^30^ Therefore, intramedullary injection might induce direct local pollution or indirect pulmonary seeding via circulation.^31^ This orthotopic model is potentially a failure for the biological research of osteosarcoma. Second, the orthotopic osteosarcoma model is not suitable for the surgical removal and corresponding investigation of bone repair. Given the osteosarcoma model was successfully established in the distal femur or proximal tibia of rats, in order to simulate the scenario clinically, we would excise the tumor first, then implant the scaffold, and conduct photothermal treatment. However, a limb‐sparing strategy is impossible for the osteosarcoma‐bearing rat. Third, although the orthotopic model of osteosarcoma has several distinct advantages including the osseous microenvironment and anatomical similarity,^32^ for immunological, pharmaceutical, and therapeutic studies, an ectopic model of osteosarcoma is sufficient due to its straightforward biological performance,^33^ which also achieved popularity in the past 30 years.^34^ In this study, we designed two independent but correlated animal models to reflect the unique property of TBGS, namely, photothermal tumor ablation in ectopic osteosarcoma‐bearing nude mice and newborn bone regeneration in rat with critical cranial defect. The data collectively supported the potential clinical value of multifunctional TBGS in the treatment of osteosarcoma.

**Figure 4:**
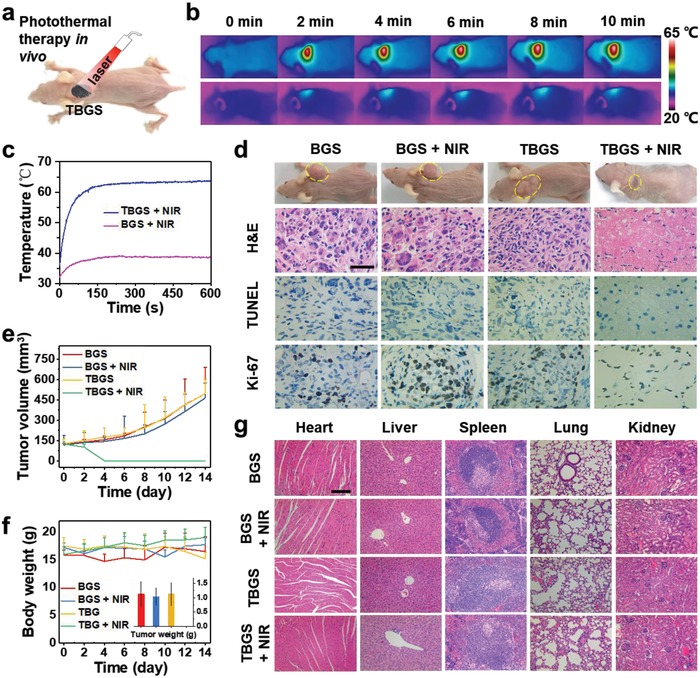
In vivo photothermal‐performance evaluation of TBGSs. a) Schematic illustration of TBGSs for in vivo photothermal cancer ablation. b) The corresponding IR thermal images at tumor sites of Saos‐2 tumor‐bearing mice in groups of BGS + laser (upper) and TBGS + laser (bottom). c) Temperature elevations at tumor sites of Saos‐2 tumor‐bearing mice in groups of BGS + laser and TBGS + laser. d) Photographs of Saos‐2 tumor‐bearing mice on 14th day after different treatments, and the tumor tissues stained by H&E, TUNEL (apoptosis), and Ki‐67 (proliferation) in 1 day after different treatments (scale bars: 10 µm). e) Time‐dependent tumor‐growth curves (*n* = 5, mean ± s.d.) after different treatments. f) Time‐dependent body‐weight curves of mice after different treatments. Inset: tumor weights of mice on the 14th day after varied treatments. *n* = 5. g) H&E staining of major organs (heart, liver, spleen, lung, and kidney) of Saos‐2 tumor‐bearing mice on the 14th day after different treatments (scale bars: 100 µm).

To evaluate the photothermal ability of TBGS, a localized in vivo photothermal tumor ablation was further assessed by employing female BALB/c nude mice bearing Saos‐2 bone tumor (**Figure** **4**a). These mice bearing Saos‐2 xenograft (subcutaneous tumor) were randomly divided into four groups (*n* = 6 for each group) for diverse treatments including BGS, BGS + NIR, 1.0 TBGS, and 1.0 TBGS + NIR, which was set based on the in vitro PTT experiments when the tumor volume reached around 120 mm^3^. Photonic tumor hyperthermia (808 nm, 1.0 W cm^−2^, 10 min) was conducted in the BGS + NIR and 1.0 TBGS + NIR groups 1 day after implanting the scaffolds into the tumor. The corresponding IR thermal images were shown at tumor sites in groups of BGS + laser and TBGS + laser (Figure 4b). As clearly shown in Figure 4c, the surface temperature of tumors implanted with 1.0 TBGSs was rapidly elevated to the equilibrium temperature of as high as 63 °C under NIR laser irradiation only within 2 min. In striking contrast, the temperature of tumors implanted with BGSs without the integrated Ti_3_C_2_ MXene only showed a slight increase to about 37 °C. As shown from the corresponding tumor photographs (2 weeks after treatment; Figure 4d), the tumors in treated groups (TBGS + NIR) were completely removed by photonic tumor hyperthermia without reoccurrence. Comparatively, the tumors in other treatment groups grew continuously without any therapeutic effect.

To further reveal the corresponding mechanism of high photothermal‐ablation efficacy, in 24 h after photothermal ablation, the necrosis of tumor tissues was qualitatively measured by hematoxylin and eosin (H&E) and terminal deoxynucleotidyl transferase‐mediated dUTP‐biotin nick end labeling (TUNEL) staining and the in vivo cellular proliferation was evaluated by Ki‐67 antibody staining (Figure 4d; Figure S9, Supporting Information). In H&E images, TBGS + NIR group was colored less blue/purple (nuclei of cells) than the control groups, namely, the number of apoptotic osteosarcoma cells in TBGS + NIR group was larger than the control groups after photothermal ablation. According to TUNEL images, more apoptotic cells (brown colors) were detected in TBGS + NIR group than that in control groups, which presented that TBGS + NIR group possessed the best therapeutic efficacy on ablating Saos‐2 tumor cells. The Ki‐67 images also manifested that the TBGS + NIR group owned the least proliferative cancer cells (dark brown), which indicated that the proliferation of Saos‐2 cancer cells was dramatically suppressed in TBGS + NIR group among the four groups and matched with the H&E and TUNEL results. The tumor volume and mice weight of the four groups were acquired every other day. Apparently, the tumor volumes of the therapeutic group represented conspicuous suppression with the final complete eradication while the tumor volume of the control groups increased rapidly (Figure 4e). Meanwhile, the body weight (Figure 4f) of all groups revealed no significant difference, implying that no obvious toxicity was induced by either pure BGSs or TBGSs.

NIR laser has limited tissue‐penetration depth. Therefore, in practical osteosarcoma removal surgery, the tumor lesion is exposed after removing the bone tumor and part of the surrounding tissues by surgery, and then TBGS are implanted. The photothermal therapy is conducted subsequently. After the photothermal therapy, the muscularis layer, subcutaneous tissue, and the skin will be successively closed. Therefore, after surgical resection, it can be considered that the bone tumor has changed from a deep tumor to a superficial tumor. In this way, the photothermal therapy can achieve the excellent therapeutic effect without the shield of soft tissues.

Subsequently, to reveal the potential acute toxicity and long‐term toxicity of composite scaffolds, we further evaluated the histocompatibility of the composite scaffolds by H&E staining of the major organs (heart, liver, spleen, lung, and kidney) of mice on the 1st, 14th, and 28th days after photothermal ablation (Figures S10 and S11, Supporting Information; Figure 4g). The H&E staining of these organ sections displayed that there was no obvious histomorphology and pathology change in these organs among the treatment group and control groups, indicating that the fabricated composite scaffolds with the integrated 2D Ti_3_C_2_ MXene have no significant acute and chronic pathological toxicity to the major organs, i.e., they are featured with high histocompatibility.

### 2.5 Ti_3_C_2_‐BG Composite Scaffolds for Stimulating Proliferation and Differentiation of hBMSCs In Vitro

**Figure 5:**
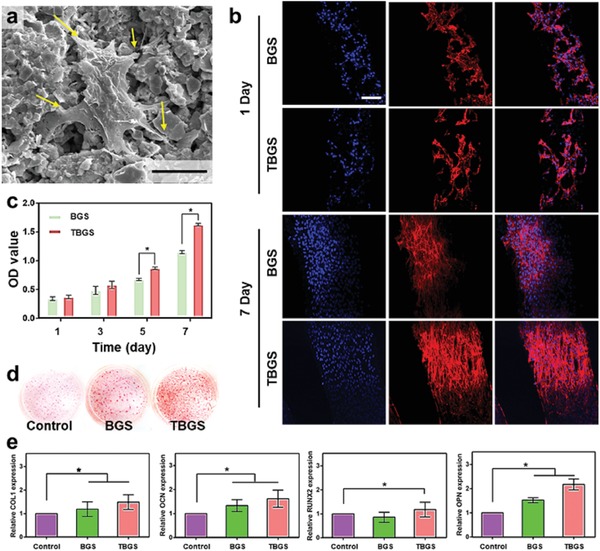
In vitro evaluation on the proliferation and osteogenic differentiation as assisted by BGS/TBGSs for bone regeneration. a) SEM image of hBMSCs after seeding on 1.0 TBGS for 1 day (scale bar: 5 µm). b) CLSM images of hBMSCs stained with DAPI (cell nuclei, blue fluorescence) and rhodamine phalloidin (cytoskeleton, red fluorescence) on BGSs/TBGSs at days 1 and 7 (scale bar: 100 µm). c) Cell proliferation as measured by a standard CCK‐8 assay at days 1, 3, 5, and 7. *n* = 3. d) Alizarin red S staining of control, BGSs, and TBGSs at day 21. e) Osteogenic gene expression (COL1, RUNX2, OPN, and OCN) of hBMSCs in control, BGS, and TBGS groups on day 7. *n* = 3. *p < 0.05, **p < 0.01, ***p < 0.001.

Bone mesenchymal stem cells (BMSCs) are able to differentiate into osteoblasts in a specific environment,^35^ therefore investigations on the in vitro adhesion and differentiation of BMSCs on TBGS have been conducted to evaluate the effect of the material on the osteogenic potential of hBMSCs. It has been found that TBGS provided hBMSCs with favorable growth environment and space, recruiting hBMSCs to adhere to its surface. In addition, hBMSCs exhibited well‐spread morphology and extended abundant pseudopods after seeding for 1 day (**Figure** **5**a; Figure S12, Supporting Information). CLSM images (Figure 5b) revealed the proliferation of hBMSCs on BGS or TBGS, which adhered to the surface of scaffolds. As compared to the BGS group, TBGS group markedly increased the proliferation of hBMSCs at day 7. Especially, hBMSCs on TBGSs exhibited abundant filopodia while the cells on BGSs had much fewer filopodia. Furthermore, the typical CCK‐8 assay also quantitatively demonstrated that TBGSs were highly biocompatible and capable of promoting cell proliferation (Figure 5c). In order to further demonstrate the bioactivity of Ti_3_C_2_ on BMSCs without bioglass, we also performed relevant experiments (Figure S13, Supporting Information). The experimental data clearly exhibited that Ti_3_C_2_ NSs with different concentrations (from 6 to 200 ppm) had no obvious toxicity to BMSCs during the evaluation period of 7 days, and the Ti_3_C_2_ NSs at low concentrations (6 ppm) even promoted the proliferation of BMSCs.

During the osteogenic differentiation of hBMSCs, calcium deposited, mineralized, and specifically formed red precipitates with Alizarin red S dye.^36^ Extracellular matrix (ECM) mineralization of hBMSCs on the control, BGSs, and TBGSs groups were estimated by Alizarin red assay. The result revealed that the number of calcium nodules was distinctly enhanced in the TBGS group at day 21 as compared to the other two groups, indicating that TBGS improved the osteogenic capability of hBMSCs in vitro (Figure 5d).

Furthermore, to evaluate the differentiation of hBMSCs in various groups (the control, BGSs, and TBGSs groups), osteoblast‐related gene expression was analyzed,^37^ including collagen type I (COL I), Runt‐related transcription factor 2 (RUNX2), osteocalcin (OCN) and osteopontin (OPN) genes. The expression of osteogenic‐specific genes in TBGSs groups was significantly enhanced at day 7 as compared to BGSs group (Figure 5e), which demonstrated that TBGS could act as the bioactive material for promoting the osteogenic differentiation of hBMSCs in vitro. All the above results further confirmed that TBGSs distinctly improved the osteogenesis of hBMSCs in vitro potentially by some titanium‐based species originating from the biodegradation productions of integrated Ti_3_C_2_ MXene, providing a promising biomaterial platform for the restoration of defective bone tissue.

### 2.6 Ti_3_C_2_‐BG Composite Scaffolds for Stimulating Osteogenic Activity In Vivo

To explore the conceivable clinical application of 3D‐printed TBGSs, the in vivo efficacy of bone regeneration of TBGSs was further assessed on Sprague–Dawley rats (SD rats) with critical cranial defect. Photothermal therapy is a potent technique for cancer therapy with minimal invasiveness and high selectivity.^38^ Meanwhile, previous results have demonstrated that short‐time NIR‐induced photothermal therapy did not impair the long‐term bone‐regeneration process.^25^ Two possible reasons are clarified as follows. On one hand, the beginning stage of the bone healing is an inflammation phase, to recruit the MSCs to the injury site.^39^ Similarly, a local inflammatory reaction will occur after photothermal treatment. On the other hand, circulating MSCs are present in the peripheral blood in minimal concentrations under normal conditions. However, their numbers significantly increase in the blood of patients with bone fracture, bone sarcomas, osteoporosis, etc. It is believed that these increased MSCs may be released from the bone marrow. In addition, previous research has demonstrated that some BMSCs involved in bone regeneration are systemically mobilized and recruited to the defective site from remote bone marrow.^40^ Therefore, we did not investigate the toxicity of NIR to local normal tissue for the bone defect repair.

**Figure 6:**
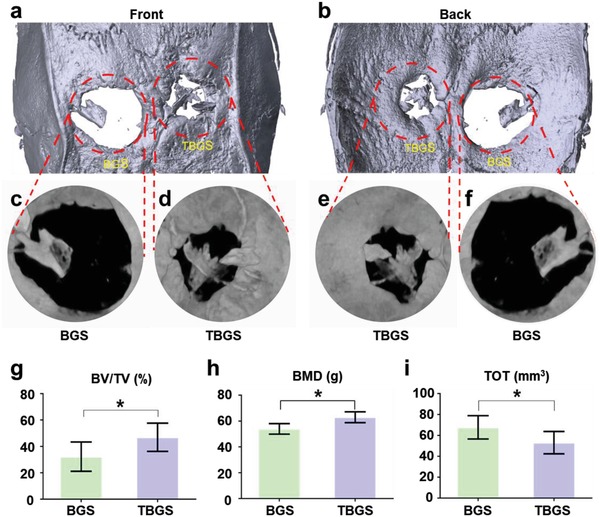
In vivo osteogenesis performance of BGS and TBGS. a,b) 3D reconstruction of circular defects at 24 weeks after scaffolds implantation. c–f) Micro‐CT images of cranial defect areas with a diameter of 5 mm at 24 weeks postoperation. g) Value of BV/TV in newborn osseous tissue (*n* = 6). h) Value of BMD in newborn osseous tissue (*n* = 6). i) Value of TOT in newborn osseous tissue (*n* = 6). *p < 0.05, **p < 0.01, ***p < 0.001.

The gross observation (3D reconstruction) and micro computed tomography (micro‐CT) analysis were conducted on samples collected at week 24 after the implantation of the composite scaffolds. 3D reconstruction of harvested craniums showed that much more calcified tissues were present in the defect implanted with TBGS, which confirmed the fact that TBGSs featured better regeneration outcome for bone defects than pure BGS without MXene integration (**Figure** **6**a,b). The micro‐CT images directly presented this result by displaying both front and back surface of a cranium that TBGS (Figure 6d,e) was more effective than BGS (Figure 6c,f) in bone regeneration at week 24. Quantitative analysis of fundamental parameters was conducted based on the histomorphometric micro‐CT analysis, such as the relative bone volume/tissue volume (BV/TV), bone mineral density (BMD), and porosity (TOT). The BV/TV, representing the percentage of newborn osseous tissue volume accounting for the entire defect space, was higher in TBGS than in BGS (Figure 6g). The BMD and TOT revealed the average bone density of circular defect areas from two perspectives (Figure 6h,i). These data collectively revealed the excellent osteogenic performance of TBGSs as compared to BGS.

**Figure 7:**
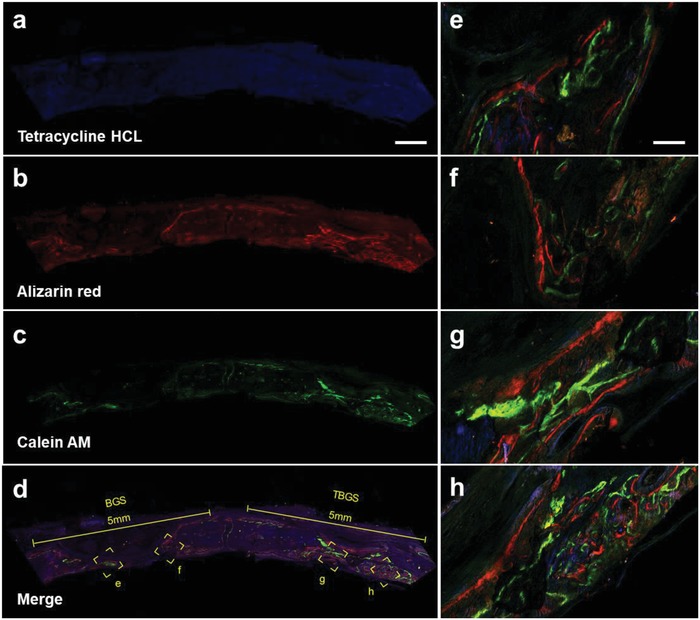
Confocal fluorescence images for superficial analysis of newborn osseous tissue of BGS and TBGS groups at week 8. a) Tetracycline HCL (blue fluorescence) injected intramuscularly into calvarial defect model rats at week 2. b) Alizarin red (red fluorescence) injected intramuscularly into calvarial defect model rats at week 4. c) Calcein‐AM (green fluorescence) injected intramuscularly into calvarial defect model rats at week 6. d) Merged image of three fluorochromes. These three fluorochromes represent newborn osseous tissue in different therapeutic duration. Scale bar in (a)–(d) is 1 mm. e,f) Magnified images represented newborn bone around BGS. g,h) Higher‐magnification images indicating the hierarchical architecture of bone around TBGS and its corresponding material‐guided regeneration process. Scale bar in images (e)–(h) is 125 µm.

Newborn osseous tissue was also further assessed by CLSM through scanning in circular defect regions. The samples of both groups were marked with tetracycline hydrochloride (HCL) (blue), Alizarin red (red), and calcein‐AM (green), and different colors represented newborn bone tissue at different stages of osteogenesis (blue fluorescence: weeks 2–4; green fluorescence: weeks 4–6; red fluorescence: weeks 6–8). The fluorescence intensity in left (BGS group) was significantly weaker than that in the right (TBGS group), which suggested that the TBGS stimulated more efficient osteogenic activity as compared with BGS (**Figure** **7**a–d). Even though both control and treatment groups were significantly stained with the three colors, the newborn osseous tissue around TBGS group (Figure 7g,h) showed better osteogenic performance compared with the BGS group (Figure 7e,f). Green and red fluorescence in the TBGS group were obviously more than that in the BGS group, indicating that more newborn osseous tissues were formed in the TBGS group than that in BGS group in the latest 4 weeks. The quantitative analysis of CLSM images (Figure S14, Supporting Information) made the osteogenesis capacities of BGS and TBGS more clearly presented, which showed that the osteogenesis rate of BGS and TBGS were about 20% and 50%, respectively. The above practices further confirmed the powerful bone reconstruct capability of TBGS in animal levels.

**Figure 8:**
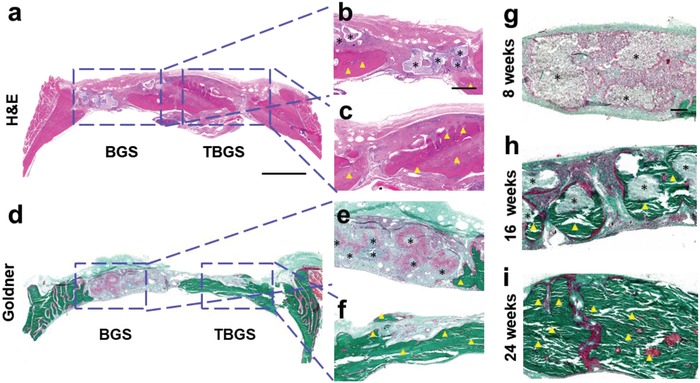
Histology staining of harvested craniums of Sprague–Dawley rats implanted with BGS/TBGS at week 24. a–c) H&E staining of harvested craniums obtained from SD rats at week 24 after operation. d–f) Goldner staining of harvested craniums of SD rats at week 24 after implanting with BGS and TBGS. g–i) Goldner staining of TBGS group at different period of weeks 8, 16, and 24. The defect areas were implanted with BGS and TBGS. Black asterisks mark implanted scaffolds that were not biodegraded completely. Yellow triangles indicate newborn osseous tissue. Scale bar in (a) and (d) is 2 mm. Scale bar in (b), (c), (e), and (f) is 500 µm. Scale bar in (g)–(i) is 200 µm.

To further evaluate the efficacy of TBGSs for bone‐defect regeneration in other aspects, the H&E staining (**Figure** **8**a–c) and Goldner staining (Figure 8d–i) were conducted. H&E staining showed that there was no inflammatory cell in either BGS or TBGS group. A large number of mineralized bone tissues (yellow triangles) was found in the bone defect implanted with TBGSs (Figure 8c). Meanwhile, there was no obviously visible residual scaffold (black asterisks) in the experiment group as compared to the BGS group (Figure 8b). Goldner staining exhibited that the defect region in the BGS group displayed a mixture of new osteoid tissue (red tissue) around the residual materials (black asterisks) (Figure 8d,e). At the same time, there was quite a lot of mineralized bone tissues (emerald green tissue) filled in the defect region of TBGS group, indicating a better newborn bone formation in TBGS group (Figure 8f). In addition, Figure 8g–i displays newborn bone‐tissue formation during different periods (weeks 8, 16, and 24) of TBGSs. Images at week 8 (Figure 8g) revealed a large amount of fibroblast and macrophage crawled in and through the pores of scaffolds. Red osteoid tissue was generated around the materials while the old scaffolds were degraded, which demonstrated the desirable simultaneous process of the degradation of old scaffolds and the formation of new osseous tissue. A lot of mineralized bone tissues were around the residual old scaffolds (Figure 8h), collectively revealing the excellent regeneration performance of TBGS. There were no obvious scaffolds left in the bone defect of TBGS group at week 24 (Figure 8i). The defect region was covered with mineralized bone, without a visible difference with the old bone tissue around the defect region. This desirable therapeutic outcome, which was attributed to effects of TBGSs, manifested the material‐guided bone regeneration process that osteoblast adhered and proliferated on the TBGSs with both osteoconduction and osteoinduction, accompanied with the formation of new osseous tissue on the vanishing scaffolds substrates. Furthermore, to evaluate the degradation of scaffolds, the BGSs and TBGSs were soaked in simulated body fluid (SBF) for 14 days at 37 °C, and the degradation rates of TBGS and BGS are ≈5% and 3%, respectively (Figure S15, Supporting Information). In addition, according to the Goldner staining images (Figure 8g–i), with the prolonging of the in vivo experiment, the residual amount of scaffold was gradually degraded. Therefore, this result proved that TBGSs owned biodegradability, high biocompatibility, and the desirable performance of accelerating tissue reconstruction.

To further investigate the in vivo long‐term toxicity (24 weeks) of BGSs/TBGSs, venous blood was collected and the major organs of rats (heart, liver, spleen, lung, and kidney) were dissected, which were fixed in a 10% formalin and stained with H&E for histological analysis after all the SD rats were executed. The hematology parameters including leucocyte, erythrocyte, hemoglobin (HGB), the percentage of neutrophil, albumin/globulin (A/G), albumin (ALB), blood urea nitrogen (BUN), cholinesterase (CHE), uric acid (URCA), K^+^, Na^+^, and Ca^2+^ were tested (Figure S16a, Supporting Information). It has been found that there were no meaningful changes in the TBGSs group in comparison to the control group. In addition, the corresponding histological sections of major organs (Figure S16b, Supporting Information) also exhibited no significant abnormalities between the control and treatment groups. Based on the above results, there were no obvious toxicity, inflammation, and infection as observed in the treated SD rats during a long therapeutic period. It also demonstrated that TBGSs were highly biocompatible for the further safe in vivo osteogenic surgery.

## 3 Conclusions

In summary, we have successfully integrated 2D Ti_3_C_2_ MXene with 3D‐pringting scaffolds for achieving simultaneous photonic bone‐tumor killing and bone‐tissue regeneration, which has been respectively demonstrated by the subcutaneous osteosarcomas model in nude mice and the bone defect model in SD rats. This composite scaffold takes the unique photothermal‐conversion performance of 2D Ti_3_C_2_ MXene and bone‐regeneration capability of BG scaffolds. The TBGS developed in this work is expected to be used for the postoperative treatment of osteosarcoma, that is, TBGSs would be implanted into the bone defect site formed by the surgical resection of bone tumor. Then, the high photothermal‐conversion performance in NIR region of TBGS would be initially used to kill the potentially residual bone‐tumor cells, and the excellent bone conduction and induction characteristics of TBGS would be then employed to repair the bone defects. As demonstrated in the experimental results, both in vitro and in vivo systematic assessments have demonstrated that these Ti_3_C_2_ MXene‐integrated composite scaffolds efficiently induced the death of bone cancer cells and eradicated the tumor on bone‐tumor xenograft by NIR irradiation. Especially and importantly, the integration of 2D Ti_3_C_2_ MXene has been demonstrated to efficiently accelerate the growth of newborn bone tissue of the composite BG scaffolds. The dual functionality of bone‐tumor killing and bone‐tissue regeneration makes these Ti_3_C_2_ MXene‐integrated composite scaffolds highly promising for the treatment of bone tumor. This first report on introducing MXene‐based nanoplatforms into tissue‐engineering biomedical field not only broadens the applications of 2D MXenes in biomedicine, but also provides an intriguing biomaterial system for initiating the related tissue engineering‐related researches.

## 4 Experimental Section

*Synthesis of Raw Ti_3_C_2_ Nanosheets*: First, the Ti_3_AlC_2_ ceramic powder was fabricated by uniformly mixing titanium powder (Alfa Aesar, Ward Hill, USA, 99.5 wt% purity; −325 mesh), aluminum powder (Alfa Aesar, Ward Hill, USA, 99.5 wt% purity; −325 mesh), and graphite powder (Alfa Aesar, Ward Hill, USA, 99 wt% purity; particle size <48 µm, −300 mesh). The powder mixture (Ti/Al/C molar ratio: 2/1/1) was ground in a planetary ball mill for 10 h and then sintered in Ar atmosphere (1500 °C, 2 h). Then, Ti_3_C_2_ NSs were synthesized by selectively removing the Al layer from the Ti_3_AlC_2_ ceramic with HF etching at room temperature according to previous report.^41^ Typically, the Ti_3_AlC_2_ powder (10 g) was immersed into HF aqueous solution (40%, 50 mL; Sinopharm Chemical Reagents Co., Ltd., Shanghai, China) in a polytetrafluoroethylene (PTFE) container, and the mixture was stirred for 48 h at room temperature. After centrifugation and washing, the precipitations were dispersed into TPAOH (50 mL, 25 wt% aqueous solution; J&K Scientific Co., Ltd., Beijing, China) under stirring for 72 h at room temperature. Finally, the resulting suspension was centrifugated and washed three times with deionized water for removing the remnant TPAOH. By this method, the raw 2D Ti_3_C_2_ NSs were obtained.^42^

*Synthesis of Raw BG Powders*: Briefly, raw BG powders were prepared via an evaporation‐induced self‐assembly (EISA) method.^43^ Typically, 80S15C BG powders (Si/Ca/P molar ratio: 80/15/5) were synthesized by dissolving tetraethoxysilane (TEOS, 53.6 g), Ca(NO_3_)_2_⋅4H_2_O (11.2 g), triethyl phosphate (TEP, 5.84 g), and HCl (0.5 m, 8 g) into ethanol (480 g). Then, the mixture was stirred at room temperature. After 24 h, the resulting sol was transferred into a petri dish for EISA process at room temperature for 7 days in a fume cupboard and then dried at 60 °C for 48 h. After being further ground, the raw BG powders were passed through 400 mesh sieve for eventually forming homogeneous size less than 37 µm.

*3D Printing of BG Scaffolds*: All printed scaffolds were fabricated by a 4th generation 3D Bioplotter (Envision GmbH, Germany). The printing ink was introduced into a polyethylene syringe tube which was fixed onto the 3D Bioplotter. A tapered nozzle (inner diameter: 400 µm) was attached to the syringe tube. Then, scaffolds (*Φ* 10 × 2 mm, pore size: 350 µm) were plotted layer by layer by extruding the paste as a fiber. The architecture was changed by plotting fibers with 0 and 60 angle steps between two successive layers. The dosing pressure to the syringe pump was 2.8–4.4 bar. The printing speed was 8–18 mm s^−1^ and the layer thickness was about 0.32 mm. Nozzle temperature was set at 30 °C and build plate temperature was consistent with the room temperature. The printed scaffolds were dried (37 °C, 12 h) and sintered (1060 °C, 3 h) to obtain pure BGSs.

*Ti_3_C_2_ MXene Integration into BG Scaffolds*: Ti_3_C_2_ NSs were suspended in distilled water by ultrasonic treatment to obtain the homogeneous Ti_3_C_2_ aqueous suspension. To prepare TBGSs, the BGSs were soaked in Ti_3_C_2_ aqueous solution at different concentrations (1.0, 1.5, and 2.0 mg mL^−1^) for 10 min and dried at 60 °C for 4 h. This operation was repeated three times and finally TBGSs were obtained. BGS integrated with 1.0 mg mL^−1^ Ti_3_C_2_ NSs was termed as 1.0 TBGS, and other TBGSs were renamed by this analogy.

*Characterization*: SEM images, EDS, and element mapping were measured on a SU8220 microscope (Hitachi, Japan). Both TEM and HRTEM images were observed by a JEM‐2100F transmission electron microscope. XPS was recorded on ESCALAB250 (Thermal Scientific, US). XRD analysis was operated on a Rigaku D/MAX‐2200 PC XRD system. Raman spectra were recorded on a high‐resolution Raman microscope (HORIBA LabRAM HR800). The CLSM images were acquired by an Olympus BX53 fluorescence microscope. NIR laser was produced using an 808 nm high‐power multimode pump laser (Shanghai Connect Fiber Optics Company). The temperature detection and thermal‐image record were conducted on an infrared thermal imaging instrument (FLIR A325SC camera, USA). The element quantitation was analyzed by inductively coupled plasma‐optical emission spectrometry (ICP‐OES, Agilent 725, Agilent Technologies, USA).

*In Vitro Photothermal Performance of Ti_3_C_2_‐BG Scaffolds*: The surface temperature of scaffolds was monitored by an infrared thermal imaging instrument. To explore the photothermal performance of different scaffolds, BGS, 1.0 TBGS, 1.5 TBGS, and 2.0 TBGS were exposed to an 808 nm laser irradiation at the power density of 1.0 W cm^−2^. Then, the photothermal performance of 1.0 TBGS at varied power densities (0.5, 0.75, and 1.0 W cm^−2^) was also investigated to explore appropriate laser power density for ablating tumor. The above experiments were conducted in a dry environment (in air). Analogously, the photothermal performance of 1.0 TBGSs at varied power densities (0.5, 0.75, and 1.0 W cm^−2^) was also assessed under wet environment (in 400 µL PBS). Finally, the photothermal stability of TBGSs was acquired (five laser “off–on” cycles, 1.0 W cm^−2^).

*In Vitro Cytotoxicity Assay and Cell Ablation*: Osteosarcoma Saos‐2 line (noted as Saos‐2 cells, Cell Bank of Shanghai Institutes for Biological Sciences, Chinese Academy of Sciences) was maintained in McCoy's 5A Medium (HyClone) and supplemented with 1% penicillin/streptomycin and 10% fetal bovine serum (FBS) in a humidified incubator (5% CO_2_, 37 °C). To investigate in vitro toxicities and anticancer effects of TBGSs, Saos‐2 cells were seeded in 48‐well plates (Corning, USA) for 24 h (1.0 × 10^5^ per well, 800 µL medium), and then the 1.0 TBGSs and BGs (*Φ* 8 mm × 1.5 mm) were gently placed on the plates to co‐incubate for additional 24 h. Afterward, the standard CCK‐8 assay was performed to quantify the cell viabilities after different treatments (*n* = 4). Different irradiation durations (0, 5, 10, and 15 min), irradiation times (0, 1, 2, and 3 times), and power densities (0, 0.5, 0.75, and 1.0 W cm^−2^) for photothermal ablation were also systematically evaluated. The OD value of wells without NIR irradiation (control, BGS, 1.0 TBGS) indicated the in vitro toxicities of materials, and the other groups (NIR, BGS + NIR, 1.0 TBGS + NIR) presented the ability of photothermal ablation against osteosarcoma cells.

To visually evaluate the photothermal‐ablation effect of scaffolds on osteosarcoma cells, Saos‐2 cells were incubated in 48‐well plates with BGSs and TBGSs. After 24 h, the BGSs and TBGSs were irradiated by 808 nm laser (10 min, 1.0 W cm^−2^). Subsequently, cells in BGSs and TBGSs with or without irradiation were stained with PI/calcein‐AM. Finally, the scaffolds were visualized by CLSM. Dead cells stained with PI showed the red fluorescence, and live cells stained with calcein‐AM exhibited the green fluorescence. All scaffolds were sterilized by UV radiation for 24 h before experimental evaluation.

*Cell Culture*: Primary hBMSCs were obtained from ScienCell Research Laboratories (the United States, #7500) and cultured with α‐MEM (Gibco) supplemented with 10% FBS (Gibco) in 5% CO_2_ at 37 °C. Every 3–4 days, the cells were detached (from the surface of the 75 cm^2^ cell culture flask (Greiner Bio‐One) using 0.25% trypsin), washed, centrifuged (1000 rpm × 5 min), resuspended (in 12 mL α‐MEM), and subcultured (in 1:3 volume ratio). Cells, from 4th to 9th generations, were used for the experiments. The cells were regularly examined under an optical microscope to monitor growth and possible contamination.

*Sample Preparation for SEM and CLSM Observation*: In short, hBMSCs (1.0 × 10^4^) were seeded in 48‐well culture plates with BGSs and 1.0 TBGSs. One day later, to observe the morphology and adhesion of hBMSCs on the scaffolds, BGSs and TBGSs were fixed with glutaraldehyde and then dehydrated with gradient concentrations of ethanol (30, 40, 50, 60, 70, 80, 90, and 100 v/v%). Then, the scaffolds with hBMSCs were observed by SEM. To further investigate the cytoskeletal change during the osteoblastic differentiation, the hBMSCs were co‐cultured with BGS or TBGS and stained by 4′,6‐diamidino‐2‐phenylindole (DAPI, blue)/rhodamine phalloidin (red) on the 1st and 7th days. Then, the CLSM photographs were recorded. Finally, BGSs and TBGSs, seeded with hBMSCs, were stained by Alizarin red to specifically mark calcium salt which was generated during the mineralization.

*Quantitative Real‐Time Polymerase Chain Reaction (QPCR) Analysis*: The effects of different scaffolds on the osteogenic differentiation of hBMSCs were assessed by measuring the mRNA expression of COL I, RUNX2, OCN, and OPN genes. The total cellular RNA was harvested with TRIzol (Invitrogen) after osteogenic induction at the 7th day. One microgram of purified RNA was then reversely transcribed into complementary DNA (cDNA) using the PrimeScript RT reagent kit (Takara, Shiga, Japan). The reverse transcription reaction was quantified by the ABI Prism 7900. Thermal Cycler used a real‐time PCR kit (SYBR Premix EX Taq, Takara, Japan). The product was quantified using a standard curve, and levels of gene expression were normalized to glyceraldehyde‐3‐phosphate dehydrogenase (GAPDH). Relative gene expression was analyzed by the 2^−ΔΔCt^ method.^44^

*Cell Toxicity and Proliferation*: To evaluate the cell toxicity and proliferation, hBMSCs were seeded on BGSs and TBGSs. Cell toxicity and proliferation were observed by CLSM after 7 days. The proliferation of cells was measured by CCK‐8 assay on the 1st, 3rd, 5th, and 7th days.

*Alizarin Red Staining*: Cells were co‐cultured with BGSs and TBGSs in 24‐transwell plates in osteogenic medium for 3 weeks. The culture medium was renewed every 2 days. After 21 days, cells were washed with PBS and fixed with 4% paraformaldehyde for 30 min at 4 °C. After that, the cells were stained with Alizarin red S solution (40 × 10^−3^ m, 2% aqueous, Sigma) for 15 min. Cells were rinsed again with PBS before being observed by microscopy.

*In Vivo Photothermal Therapy in NIR Biowindow*: Female BALB/c nude mice (about 13 g) were subcutaneously injected with Saos‐2 cells (4 × 10^6^ cell per site) to establish the ectopic osteosarcoma model. When the volume of the tumors reached about 120 mm^3^, the mice were divided into four groups including BGS group, BGS + NIR laser group, TBGS group, and TBGS + NIR laser group (*n* = 6 in each group). Then, a small incision was carefully made to expose the tumor, and the scaffolds (BGSs or TBGSs, 8 mm × 1.5 mm × 1.5 mm) were implanted into the center of the lesion, and the subsequent surgical sutures were used to close the wound. Twenty‐four hours later, the in vivo photothermal therapy was performed. Laser irradiation was carried out on the BGS + NIR laser group and TBGS + NIR laser group. Each mouse was anesthetized and exposed to the 808 nm laser for 10 min (1.0 W cm^−2^). The tumor surface temperature and the thermal images of mice were recorded by an infrared thermal camera during the treatment. The NIR treatment time was set as day 0. From day 0, the tumor volume and the body weight of all mice were monitored every 2 days during half a month after the corresponding treatments. The tumor volume was calculated according to the following formula: tumor volume (*V*) = (tumor length) × (tumor width)^2^/2 − scaffold volume. The tumors were dissected and sectioned into slices to qualitatively measure the necrosis. The tumor slices were stained with H&E, TUNEL, and Ki‐67 antibody. To further investigate in vivo toxicity of pure BGSs and TBGSs, the major organs (heart, liver, spleen, lung, and kidney) of four groups of mice were obtained and stained with H&E on the 1st, 14th, and 28th days, respectively.

*Animal Surgical Procedures*: All surgical procedures were performed on 8 week old male SD rats. Following anesthesia with intraperitoneal pentobarbital (5 mg/100 g; Sigma), two 5 mm defects in the frontal‐parietal bone were created using an electric trephine (Nouvag AG, Goldach, Switzerland). After that, the calvarial defects were filled with BGS and TBGS, respectively (*Φ* 5 mm × 2 mm). Finally, the incision was closed by suturing the periosteum and skin separately. The HCL, Alizarin red, and calcein‐AM were injected intramuscularly at weeks 2, 4, and 6. Rats were successively killed by an overdose of anesthetic after 8, 16, and 24 weeks. Craniums were gathered and fixed in a 4% paraformaldehyde solution overnight before further analysis.

*Micro‐CT Analysis*: All the harvested specimens were examined using the mCT‐80 system to evaluate new bone formation within the defect region. The undecalcified samples were scanned at a resolution of 18 µm. After 3D reconstruction, the relative BV/TV, BMD, and total porosity (TOT) in the defect regions were used to calculate new bone formation using the auxiliary software of the mCT‐80 system44.

*Histological Analysis of Newborn Osseous Tissue*: After decalcification and paraffin embedding, specimens were cut into 5 µm thick sections and then incubated at 60 °C for 1.5 h. To evaluate the newborn osseous tissues around the BGS/TBGS, hard tissue slices were stained with H&E and Goldner's trichrome method. For Goldner staining, sections were placed in Weigert's Hematoxylin for 30 min, washed in running tap water for 10 min, and then stained in Ponceau Acid Fuchsin, phosphomolybdic acid–Orange G solution, and Light Green stock solution. Photomicrographs were acquired using a LEICA DM 4000. Meanwhile, standard blood tests were also performed.

*Statistical Analysis*: All data were reported as mean ± standard deviation. Statistical comparisons were conducted with Student's two‐sided *t*‐test as *p < 0.05 (statistically significant), **p < 0.01 (moderately significant), and ***p < 0.001 (highly significant).

## Conflict of Interest

The authors declare no conflict of interest.

## Supporting information

Supporting Information
