# Supplementary material for: 2D MXene‐Integrated 3D‐Printing Scaffolds for Augmented Osteosarcoma Phototherapy and Accelerated Tissue Reconstruction
Source: Adv Sci (Weinh). 2019 Nov 26;7(2):1901511. doi: 10.1002/advs.201901511 (PMC6974945; doi:10.1002/advs.201901511)
Supplement: Supplementary file 1 — Supporting Information [file ADVS-7-1901511-s001.pdf]

Copyright WILEY-VCH Verlag GmbH & Co. KGaA, 69469 Weinheim, Germany, 2019.

ADVANCED SCIENCE
Open Access

# Supporting Information

for *Adv. Sci.,* DOI: 10.1002/advs.201901511

# 2D MXene-Integrated 3D-Printing Scaffolds for Augmented Osteosarcoma Phototherapy and Accelerated Tissue Reconstruction

*Shanshan Pan, Junhui Yin, Luodan Yu, Changqing Zhang, Yufang Zhu,* Youshui Gao,* and Yu Chen**

# Supporting information for

## 2D MXene-Integrated 3D-Printing Scaffolds for Augmented Osteosarcoma Phototherapy and Accelerated Tissue Reconstruction

*Shanshan Pan[1], Junhui Yin[1], Luodan Yu, Changqing Zhang, Yufang Zhu*, Youshui Gao* and Yu Chen**

S. Pan, Dr. L. Yu, Prof. Y. Zhu, Prof. Y. Chen
State Laboratory of High Performance Ceramics and Superfine Microstructure, Shanghai Institute of Ceramics, Chinese Academy of Sciences, Shanghai, 200050, P. R. China.
E-mail: zhuyufang@mail.sic.ac.cn; chenyu@mail.sic.ac.cn

S. Pan, Prof. Y. Zhu
School of Materials Science and Engineering, University of Shanghai for Science and Technology, Shanghai 200093, P. R. China.

Dr. J. Yin, Prof. C. Zhang, Prof. Y. Gao
Department of Orthopedic Surgery, Shanghai Jiao Tong University Affiliated Sixth People's Hospital, Shanghai 200233, P. R. China.
E-mail: gaoyoushui@sjtu.edu.cn

[1]These authors contributed equally to this work.

# Supplementary figures

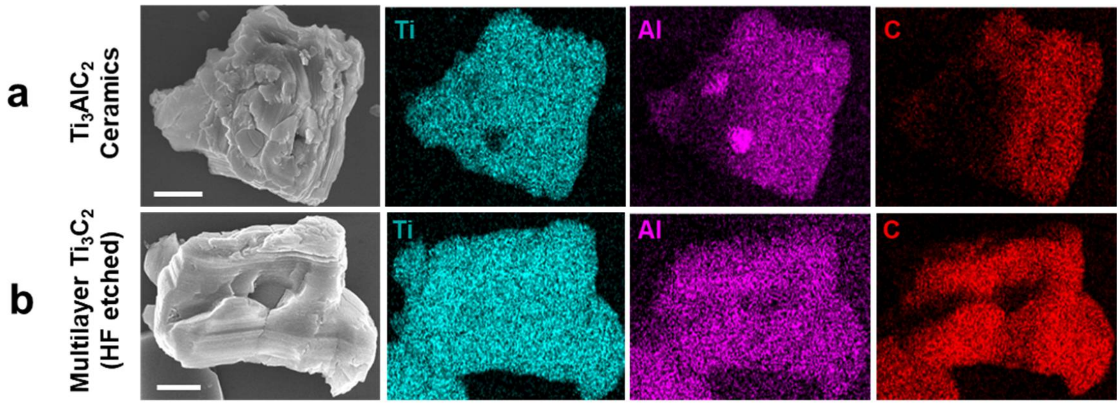


**Supplementary Figure S1.** Elemental mapping for $Ti_3AlC_2$ ceramic and multilayer $Ti_3C_2$, scale bar is 1 μm.

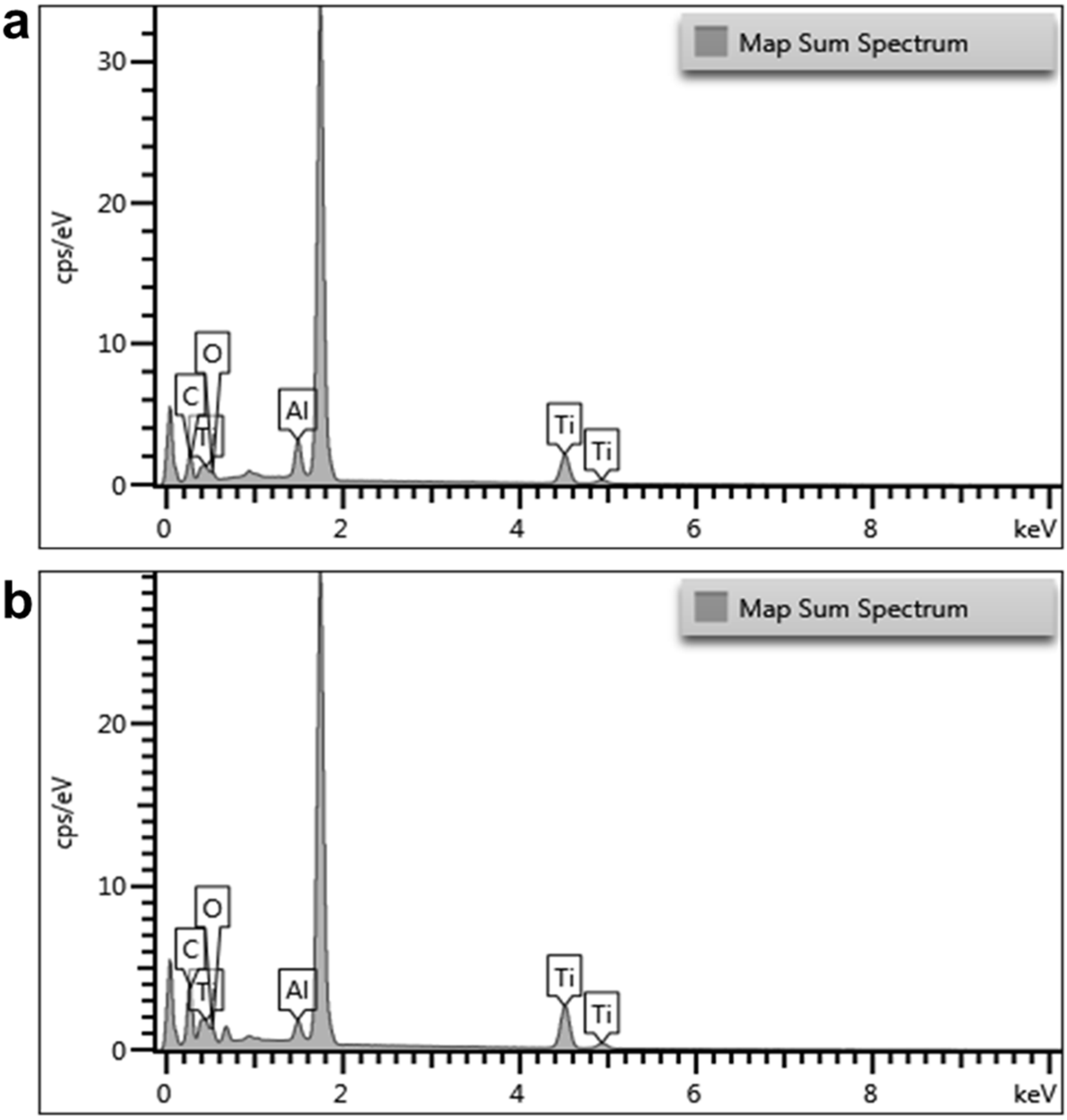


**Supplementary Figure S2. (a)** EDS profile confirming the presence of Ti, Al and C elements in $Ti_3AlC_2$ ceramic on **Supplementary Figure S1**. The atomic content of Ti, Al and C elements are 44.3%, 14.8% and 34.0%, respectively. **(b)** EDS profile confirming the presence of Ti, Al and C elements in HF-etched $Ti_3AlC_2$ (multilayer $Ti_3C_2$) on **Supplementary Figure S1**. The atomic percent of Ti, Al and C elements are 40.5%, 5.5% and 43%, respectively.

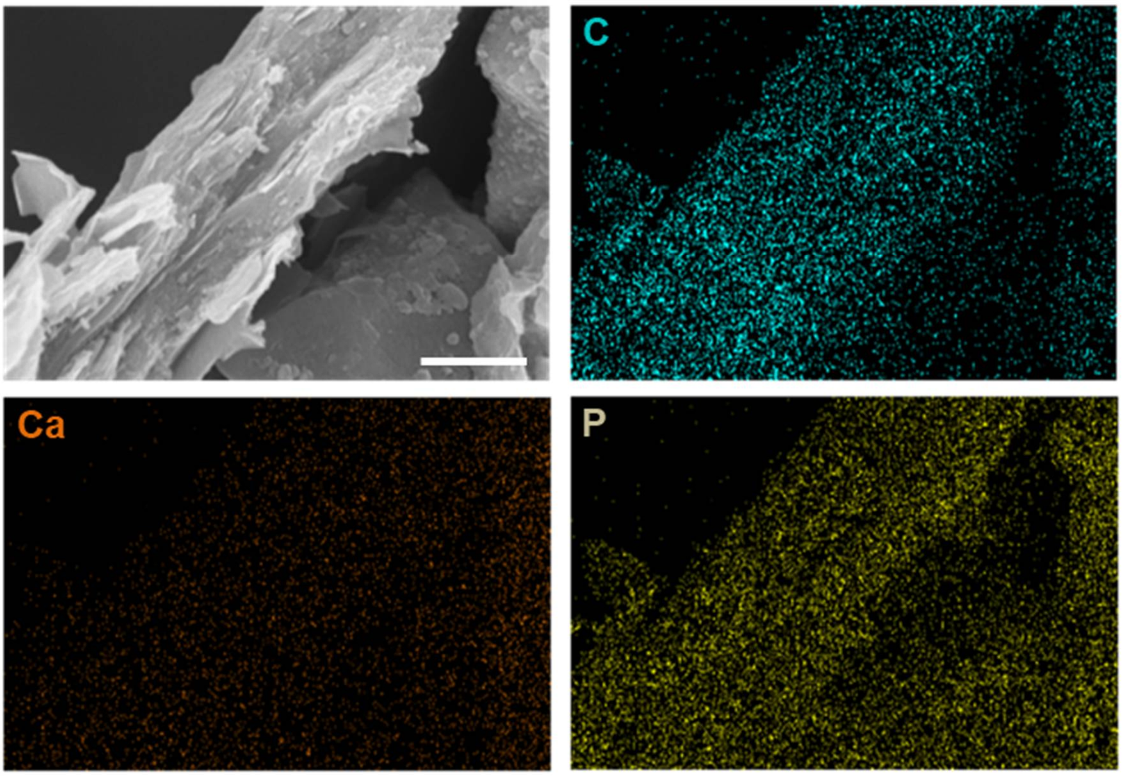


**Supplementary Figure S3.** SEM image and the corresponding elemental mapping for cross-section of TBGSs (scale bar: 1 μm).

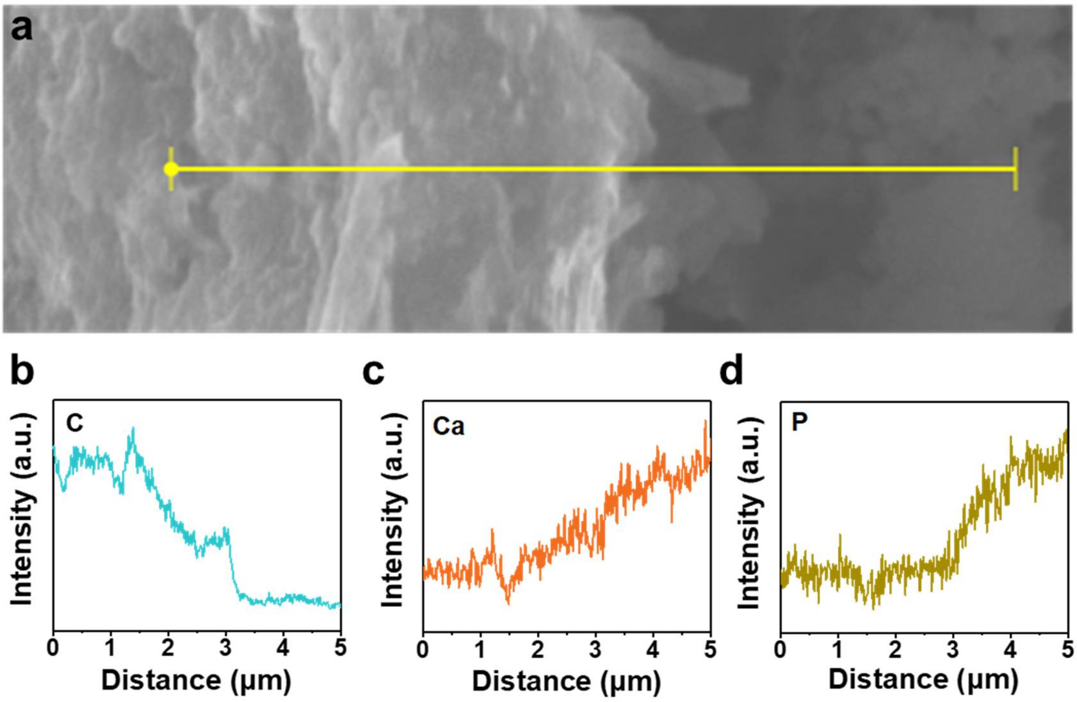


**Supplementary Figure S4.** EDS linear scanning of the interface of TBGSs for C, Ca, and P elements.

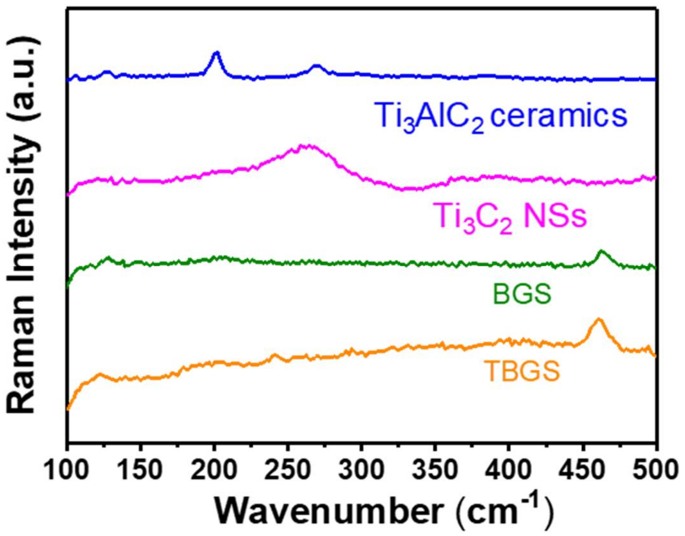


**Supplementary Figure S5.** Raman spectra of $Ti_3AlC_2$ bulk ceramic, $Ti_3C_2$ NSs, pure BGSs and TBGSs.

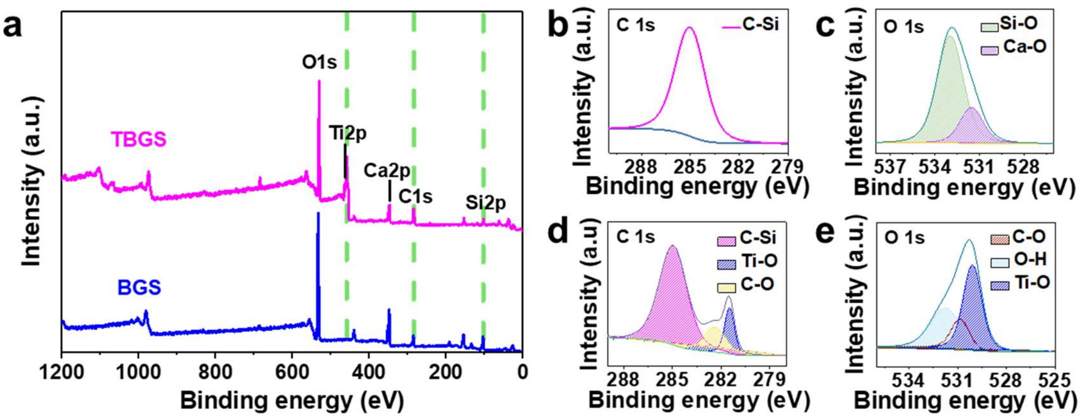


**Supplementary Figure S6. (a)** XPS spectra of pure BGS and TBGS in the area including all possible elements. **(b)** C 1s peak of pure BGSs. **(c)** O 1s peak of pure BGSs. **(d)** C 1s peak of TBGSs. **(e)** O 1s peak of TBGSs.

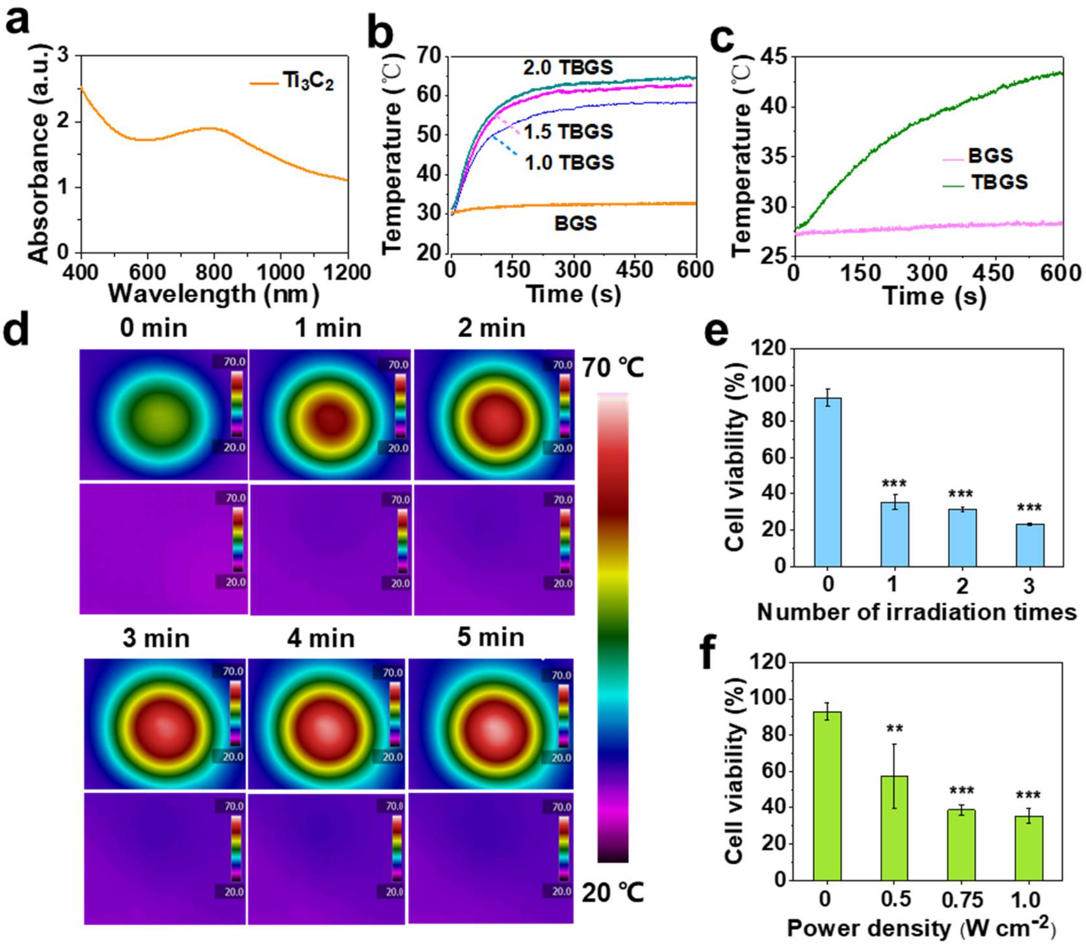


**Supplementary Figure S7. (a)** UV-vis-NIR spectrum of $Ti_3C_2$ NSs. **(b)** Photothermal heating curves in air of pure BGS and TBGSs. **(c)** Photothermal heating curves in PBS of pure BGS and TBGSs. **(d)** Thermal images of BGS and 1.0 TBGSs irradiated by 808 nm laser at the power density of 1.0 W $cm^{-2}$. **(e)** Relative cell viability of Saos-2 cells treated with 1.0 TBGSs for different irradiation durations. n = 4. **(f)** Relative cell viability of Saos-2 cells treated with 1.0 TBGSs for different power densities. n = 4. **p < 0.01, *** p < 0.001.

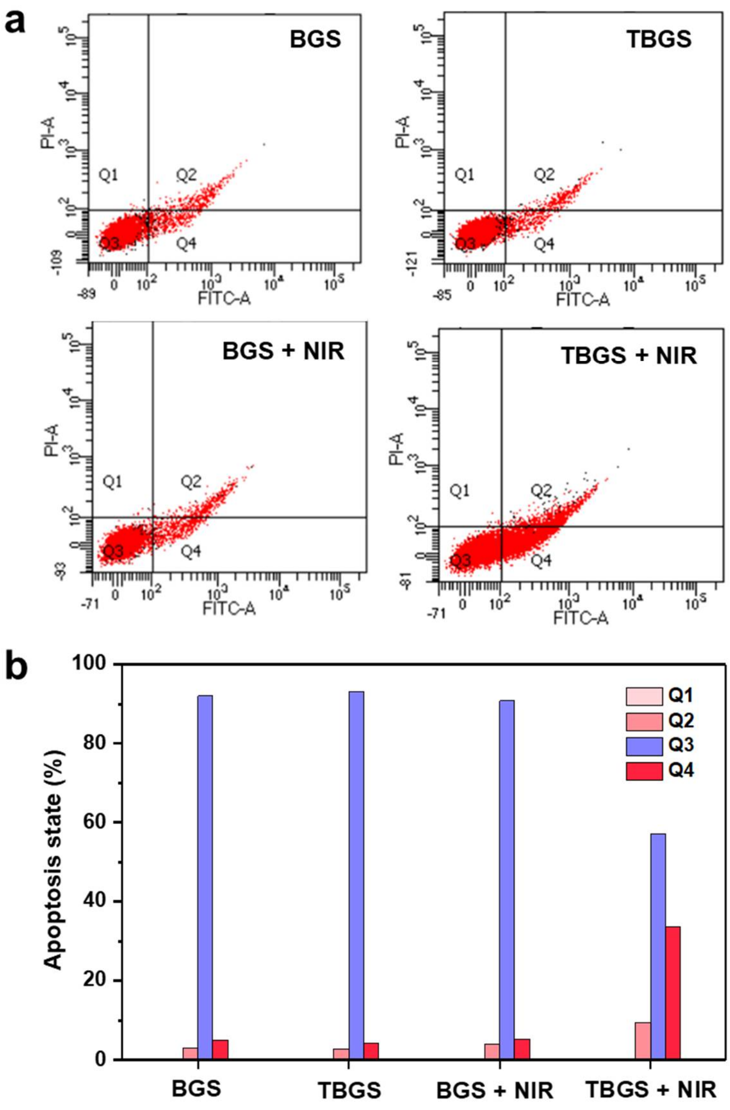


**Supplementary Figure S8. (a)** Apoptosis flow cytometry assay of Saos-2 cells on BGSs/TBGSs with/without laser irradiation. Region Q1, Q2, Q3, Q4 represent dead cells, late apoptotic cells, live cells, and early apoptotic cells, respectively. **(b)** Corresponding quantitative analysis of Saos-2 cells at different stages. In BGS group, the number of cells in region Q1, Q2, Q3, Q4 is 0.1%, 3.0%, 92% and 4.9%, respectively. In TBGS group, the number of cells in region Q1, Q2, Q3, Q4 is 0.0%, 2.8%, 93% and 4.2%, respectively. In BGS + NIR group, the number of cells in region Q1, Q2, Q3, Q4 is 0.1%, 4.0%, 90.8% and 5.2%, respectively. In TBGS + NIR group, the number of cells in region Q1, Q2, Q3, Q4 is 0.1%, 9.4%, 57% and 33.5%, respectively.

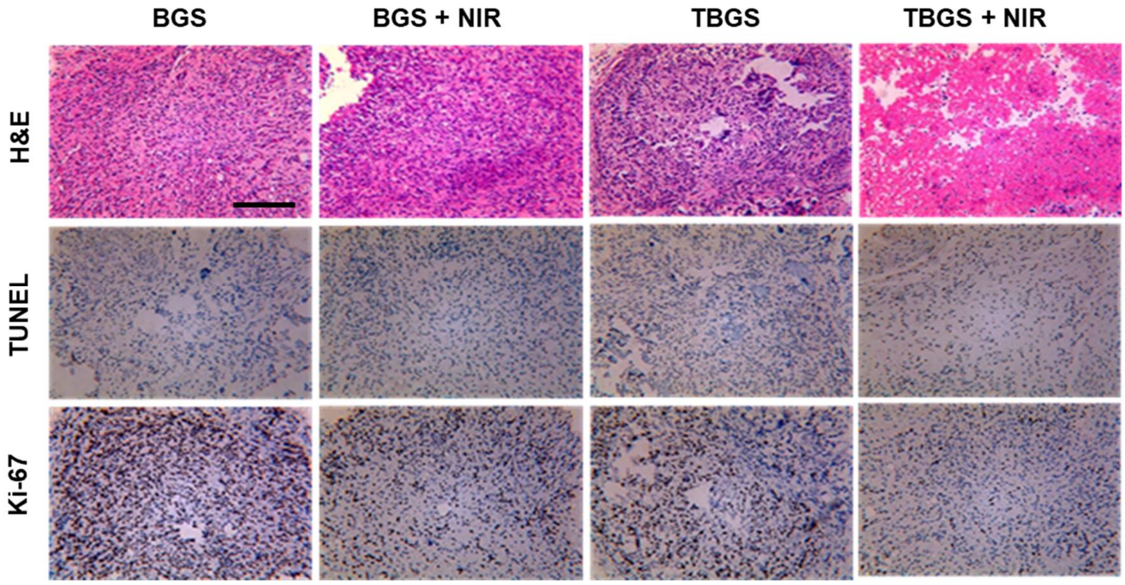


**Supplementary Figure S9.** Histological sections of tumors after different treatments at the 1st day (scale bar: 100 μm).

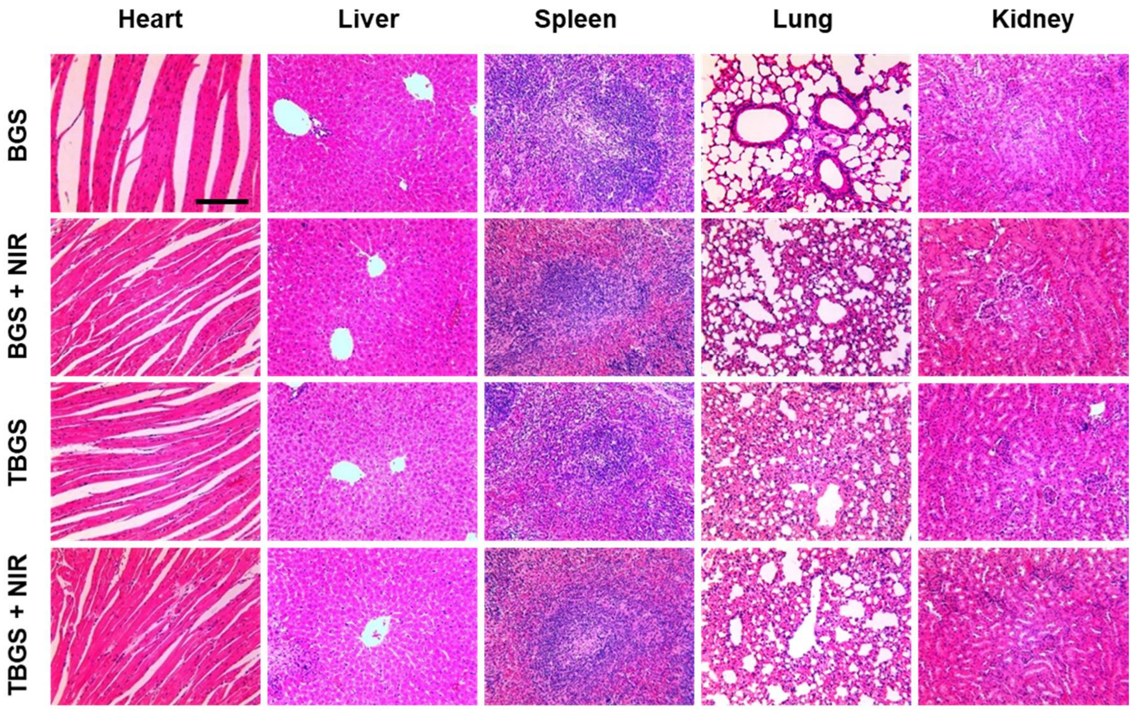


**Supplementary Figure S10.** Histological observation of major organs from mice bearing tumors after different treatments at the 1st day (scale bar: 100 μm).

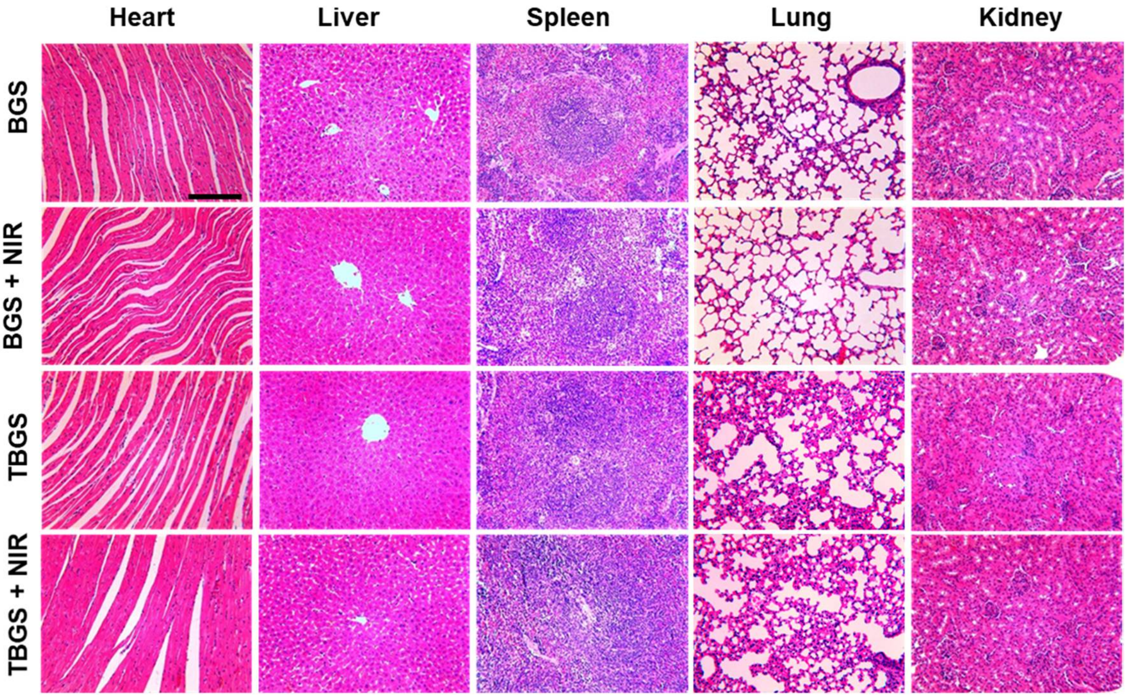


**Supplementary Figure S11.** Histological observation of major organs from mice bearing tumors after different treatments at the 28th day (scale bar: 100 μm).

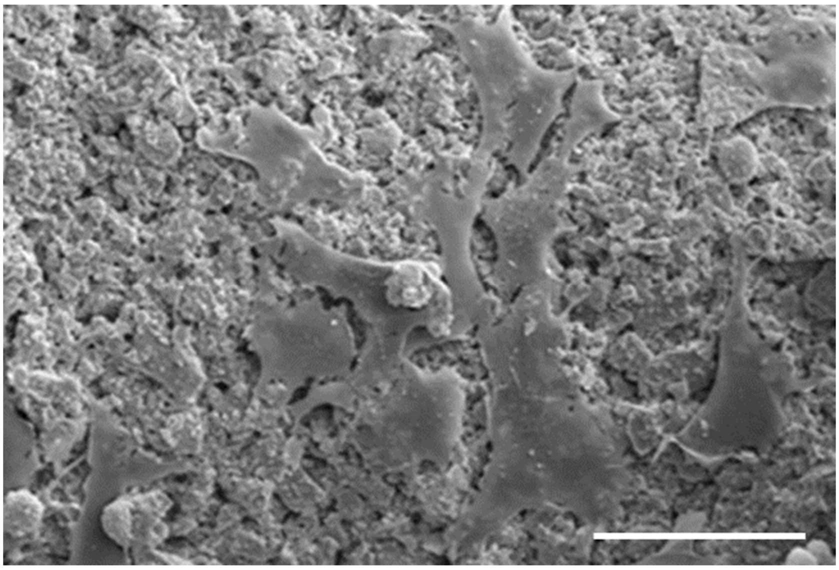

**Supplementary Figure S12.** SEM image of hBMSCs after seeding on 1.0 TBGS for 1 day (scale bar: 50 μm).

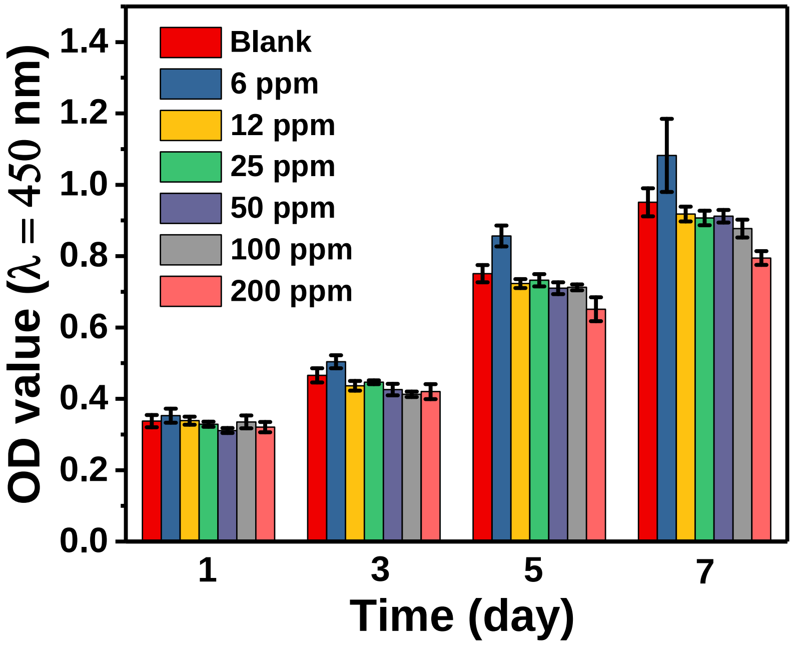


**Supplementary Figure S13.** CCK-8 data of BMSCs incubated with $Ti_3C_2$ NSs for 7 days (n = 4).

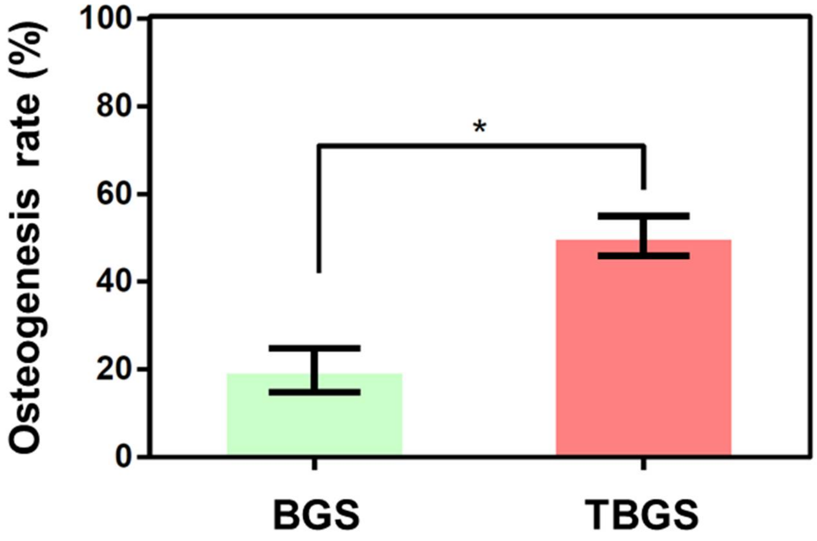


**Supplementary Figure S14.** The osteogenesis rate of BGSs and TBGSs. The graph shows the percentage of each fluorochrome area for different groups in **Figure 7** (n = 4, $*p < 0.05$).

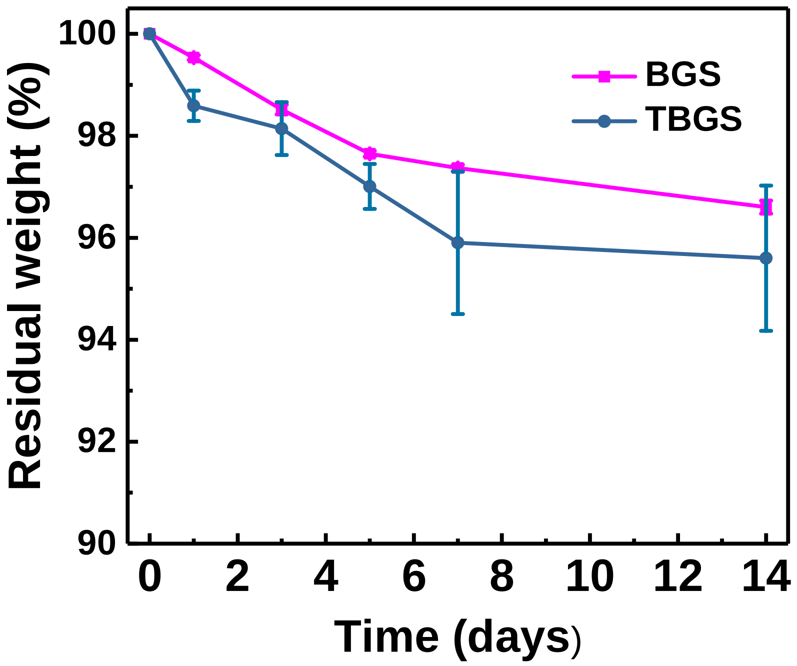


**Supplementary Figure S15.** Degradation of BGSs and TBGSs soaked in simulated body fluid for 14 days at 37 □ (n = 3).

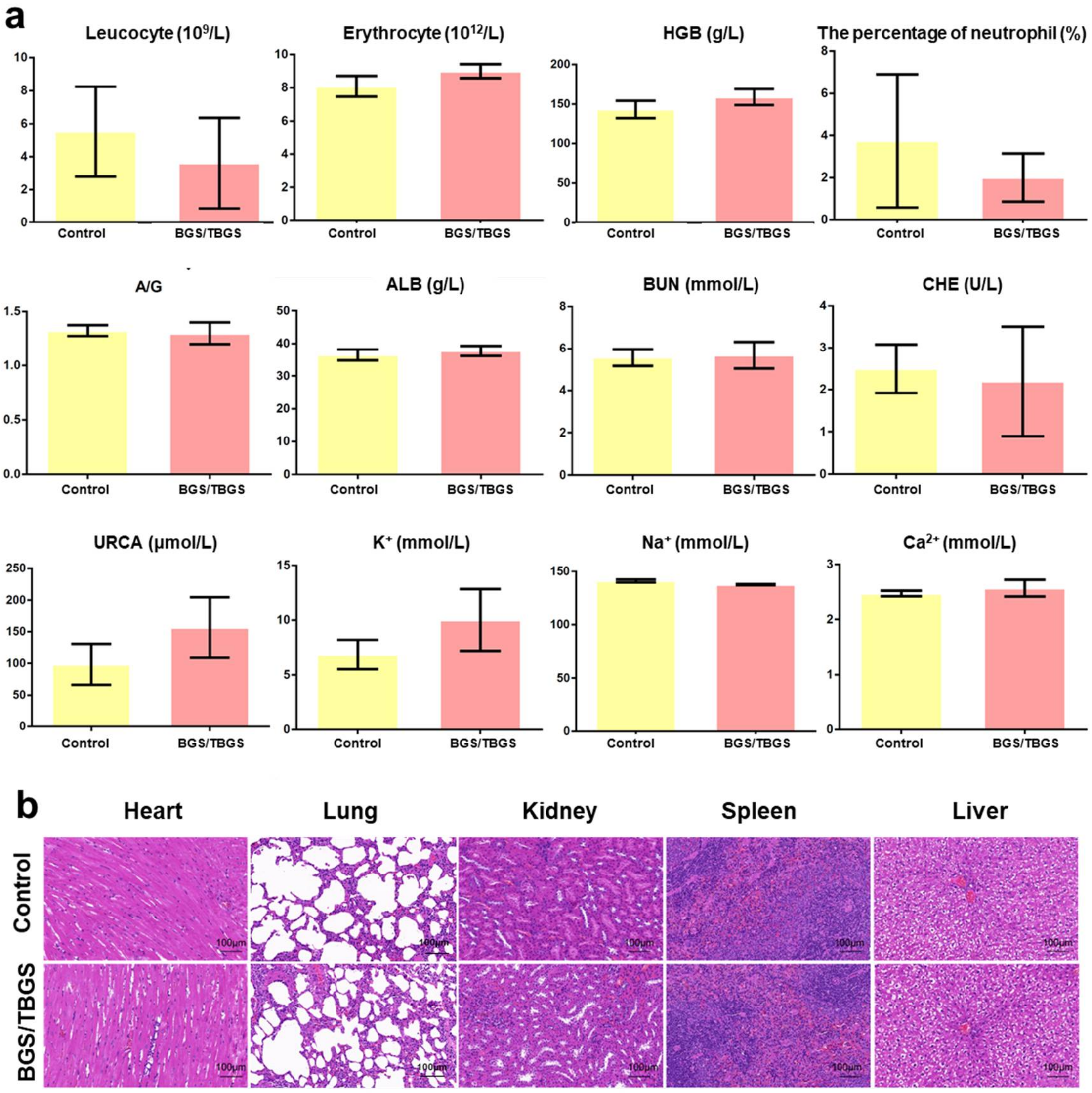


**Supplementary Figure S16. (a)** Hematological assay of SD rats implanted with BGS/TBGS compared to the control group. n = 6. **(b)** H&E stain of major organs of BGS/TBGS group and control group. There were no significant differences between the two groups at week 24 (scale bar: 100 μm).
